# The Evolving Phenotype of Myocarditis: The Role of Cardiovascular Magnetic Resonance from Acute Injury to Healing and Residual Scar

**DOI:** 10.3390/jcm15218352

**Published:** 2026-10-27

**Authors:** Carlotta Rossignoli, Roberto Scarsini, Giorgia Alberti, Giovanni Benfari, Leonardo Portolan, Martina Magistri, Alessia Gambaro, Flavio Ribichini, Anna Piccoli

**Affiliations:** 1 Division of Cardiology, Department of Medicine, University of Verona, Piazzale Aristide Stefani, 1, 37126 Verona, Italy; rossignolicarlotta@gmail.com (C.R.);; 2 Department of Clinical and Molecular Medicine, Sapienza University, Via Giorgio Nicola Papanicolau, 00189 Rome, Italy

**Keywords:** myocarditis, cardiac magnetic resonance, myocardial inflammation, tissue characterization, parametric mapping, longitudinal imaging

## Abstract

Myocarditis is a dynamic disease, and myocardial oedema, injury, ventricular dysfunction, and fibrosis may evolve differently over time. Cardiovascular magnetic resonance (CMR) has become central to its non-invasive assessment, providing complementary information on myocardial tissue through T2-sensitive imaging, native T1, extracellular volume (ECV), and late gadolinium enhancement (LGE). The meaning of these findings, however, depends on when imaging is performed. Follow-up CMR may therefore provide a better understanding of myocardial recovery, helping to characterize resolving myocardial injury, persistent tissue abnormalities, and residual post-acute changes. Based on the temporal evolution of CMR findings, we propose four conceptual patterns of myocardial recovery: near-complete imaging recovery; resolving oedema/injury-pattern abnormalities; persistent oedema/injury-pattern abnormalities; post-acute injury with residual LGE and potential structural remodelling. These patterns are intended as a framework for interpreting serial CMR rather than as formally validated imaging phenotypes, and should be considered complementary to contemporary clinical disease-stage and mechanistic classifications. In selected patients, integration with echocardiography, molecular imaging, genetics, and endomyocardial biopsy may provide further insight into the underlying disease process. Whether this longitudinal approach can improve risk stratification and ultimately guide therapeutic decisions remains an important question for future studies.

## 1. Introduction

Myocarditis is an inflammatory disease of the myocardium that may result from infectious agents, exposure to toxic substances, or immune-mediated mechanisms. Its clinical course is highly variable: while most cases resolve spontaneously, myocarditis may occasionally present with severe myocardial injury or sudden cardiac death and, in a subset of patients, persistent inflammation and myocardial scarring may lead to adverse ventricular remodelling and eventually to dilated or hypokinetic non-dilated cardiomyopathy. Acute myocarditis generally refers to disease presenting within one month of symptom onset, whereas chronic inflammatory cardiomyopathy is characterized by persistent myocardial inflammation in the setting of an established cardiomyopathic phenotype [1].

The 2025 ESC Guidelines introduced a revised conceptual framework for myocarditis and pericarditis, recognizing these conditions as part of a broader spectrum termed inflammatory myopericardial syndrome (IMPS). IMPS is proposed as an umbrella term during the initial diagnostic work-up, encompassing isolated myocarditis and pericarditis as well as overlapping forms, including myopericarditis and perimyocarditis, until a more specific diagnosis is established [2]. This approach reflects the shared inflammatory nature, overlapping clinical presentations, and frequent coexistence of myocardial and pericardial involvement, moving beyond the traditional view of these conditions as separate entities. Importantly, the new framework also emphasizes a clinically driven diagnostic pathway in which multimodality cardiovascular imaging, particularly cardiovascular magnetic resonance (CMR), plays a central role in the non-invasive identification of myocardial inflammation and in defining the predominant pattern of cardiac involvement. Within this framework, imaging is not merely supportive of the diagnosis but contributes to phenotypic characterization by defining the presence, distribution, and extent of myocardial and pericardial involvement. However, CMR findings should not be considered diagnostic of myocarditis in isolation, as tissue abnormalities such as myocardial oedema, increased native T1 or T2, and non-ischaemic late gadolinium enhancement (LGE) are not pathognomonic and may occur in other myocardial diseases. Accordingly, the diagnosis of myocarditis remains an integrated clinical diagnosis, requiring CMR findings to be interpreted in conjunction with the clinical presentation, biomarkers, ECG and, in selected cases, endomyocardial biopsy and other diagnostic investigations. The IMPS concept therefore reinforces the need to interpret imaging findings as part of a continuous and potentially evolving inflammatory spectrum rather than within rigid diagnostic categories. This paradigm is particularly relevant to longitudinal imaging, as the relative contribution of myocardial inflammation, pericardial involvement, ventricular dysfunction, and residual structural abnormalities may change throughout the course of the disease.

Historically, much of the diagnostic framework for myocarditis has been organized around a binary question on the presence or absence of myocardial inflammation. This approach is understandable because establishing the diagnosis remains challenging. Endomyocardial biopsy (EMB) provides unique histological, immunohistochemical, and potentially etiological information but is not required in every patient and remains particularly important in high-risk presentations or when the identification of a specific histological subtype may have a therapeutic impact [1,2,3]. CMR, in contrast, has emerged as the principal non-invasive technique for myocardial tissue characterization and plays a central role in the assessment of clinically suspected myocarditis. Accordingly, the 2025 ESC Guidelines recommend CMR in patients with clinically suspected myocarditis for the non-invasive diagnosis of myocardial inflammation using the updated Lake Louise Criteria (Class I, Level of Evidence B); within this framework, CMR-proven myocardial involvement in an appropriate clinical presentation can establish a definite clinical diagnosis of myocarditis, while tissue characterization and the spatial distribution of myocardial injury contribute to distinguishing inflammatory injury from alternative causes of acute myocardial damage [2].

The Lake Louise Criteria (LLC), first proposed in 2009, established a CMR-based approach to the diagnosis of myocarditis using three main features of myocardial inflammation: oedema, hyperaemia and increased capillary permeability, and non-ischaemic myocardial injury identified by LGE. The criteria were subsequently revised in 2018 to incorporate quantitative tissue characterization with parametric mapping, improving the ability of CMR to detect myocardial inflammatory changes. According to the updated LLC, CMR findings support acute myocardial inflammation when at least one T2-based criterion, reflecting increased myocardial water content, is combined with at least one T1-based criterion indicating myocardial injury. This approach therefore integrates complementary information on the two major components of acute myocarditis: inflammatory oedema and tissue damage [2,4]. An improved ability of CMR to characterize acute myocarditis and a reinforced role in contemporary diagnostic algorithms were both achieved.

Besides CMR, other imaging modalities may provide useful information in patients with suspected myocarditis. Transthoracic echocardiography is generally the first imaging examination performed, although its diagnostic specificity is limited. Findings range from a completely normal examination to regional wall motion abnormalities, global left ventricular dysfunction, increased wall thickness related to myocardial oedema, right ventricular involvement, and pericardial effusion. Echocardiography also has an important role in the assessment of haemodynamic status and in monitoring changes in ventricular function during the acute phase. Speckle-tracking echocardiography may provide additional information, as impaired global longitudinal strain can be detected even when left ventricular ejection fraction is preserved [5].

Cardiac computed tomography (CT) is particularly useful in patients presenting with chest pain and elevated troponin levels, in whom coronary artery disease needs to be excluded. In appropriately selected patients, coronary CT angiography provides a non-invasive alternative to invasive coronary angiography and may help exclude obstructive coronary artery disease in selected patients with an infarct-like presentation. Cardiac CT can also provide information on left ventricular function, including the presence of global or regional wall motion abnormalities. More recently, advances in CT technology have expanded its potential beyond coronary and functional assessment. Late iodine enhancement and CT-derived extracellular volume quantification allow myocardial tissue characterization and may provide additional information on myocardial injury. However, despite promising initial results, the clinical role of these techniques in patients with suspected myocarditis remains to be fully established [6,7,8].

^18^F-fluorodeoxyglucose positron emission tomography (FDG-PET) provides a complementary approach to the assessment of myocardial inflammation by detecting increased glucose metabolism in activated inflammatory cells. Although FDG-PET is well established in the evaluation of cardiac sarcoidosis, its role in the routine diagnostic work-up of uncomplicated myocarditis remains more limited [2,9,10,11,12]. Importantly, limitations of CMR that may favour the use of FDG-PET should be distinguished from limitations intrinsic to FDG-PET itself. Irregular heart rhythms may impair CMR image acquisition and quality, while cardiac implantable electronic devices may generate susceptibility-related artefacts that interfere with CMR myocardial tissue characterization. In such circumstances, FDG-PET may provide an alternative approach to the assessment of myocardial inflammatory activity when CMR is unsuitable or diagnostically limited [2]. By contrast, the principal limitations of FDG-PET relate to the need to suppress physiological myocardial glucose uptake and to the technical characteristics of PET/CT acquisition. Reliable assessment of myocardial inflammatory activity requires strict patient preparation, typically including dietary carbohydrate restriction followed by prolonged fasting; inadequate suppression of physiological myocardial FDG uptake may produce diffuse or heterogeneous myocardial activity and substantially reduce diagnostic interpretability [2,9,10,11,12]. In addition, PET/CT may itself be affected by metal-related artefacts from cardiac implantable electronic devices, particularly through CT-based attenuation correction; however, these should be distinguished from the magnetic-susceptibility artefacts affecting CMR. Additional limitations of FDG-PET include ionizing radiation exposure, limited standardization, and more limited evidence supporting its routine use in uncomplicated myocarditis [2,9,10,11,12].

The complementary roles, strengths, and limitations of cardiovascular imaging modalities in myocarditis are summarized in Table 1.

However, the diagnostic phase represents only the beginning of the disease trajectory.

Myocarditis is an intrinsically dynamic disease, in which inflammatory infiltration, myocardial oedema, myocyte injury, necrosis, and subsequent repair and fibrosis evolve over time and at different rates. Importantly, clinical and biochemical recovery do not necessarily parallel resolution of myocardial abnormalities, as CMR signs of myocardial injury may persist despite normalization of symptoms and cardiac biomarkers. Likewise, recovery of left ventricular systolic function does not invariably imply complete tissue healing, and residual LGE may remain detectable after resolution of myocardial oedema. Conversely, both oedema and LGE observed during the acute phase may substantially decrease over the following weeks or months, highlighting the importance of the timing of imaging assessment and, when clinically indicated, serial CMR evaluation. Consequently, CMR findings should be interpreted in relation to the timing of imaging, as they reflect a disease process that evolves over time [18,19,20].

These observations support a longitudinal approach to cardiovascular imaging in myocarditis, as the significance of imaging findings changes throughout the course of the disease. At presentation, the main imaging goal is to identify findings compatible with active myocardial inflammation.

In this review, we examine myocarditis from this longitudinal imaging perspective, focusing on CMR as the key imaging modality and on the transition from active inflammation to resolving injury, persistent abnormalities, and potential structural remodelling. We also discuss the implications of these findings for prognosis, arrhythmic risk stratification, follow-up, return to exercise, and imaging-guided management.

In defining this longitudinal perspective, however, it is important to acknowledge the population that currently provides the strongest supporting evidence. The present review focuses primarily on adult myocarditis, and the proposed CMR trajectories are informed largely by studies of adults with acute, clinically uncomplicated myocarditis, frequently presenting with an infarct-like phenotype.

Paediatric myocarditis represents a distinct and age-dependent clinical spectrum. Infants and younger children may more frequently present with non-specific symptoms, heart failure, and ventricular dysfunction, whereas adolescents may exhibit a more adult-like infarct-like presentation with chest pain and troponin elevation [21]. Although CMR is increasingly used for diagnosis and risk stratification in children, the available evidence remains more limited than in adults, particularly with regard to serial multiparametric CMR assessment [22]. Accordingly, the longitudinal imaging patterns proposed in this review should not be directly extrapolated to paediatric myocarditis and require dedicated validation in paediatric cohorts.

## 2. Literature Search and Evidence Selection

This narrative review was informed by a targeted literature search of PubMed, performed up to September 2026. Search concepts included combinations of terms related to “myocarditis”, “cardiovascular magnetic resonance”, “cardiac magnetic resonance”, “CMR”, “follow-up”, “serial imaging”, “T1 mapping”, “T2 mapping”, “late gadolinium enhancement”, “extracellular volume”, “myocardial oedema”, and “scar”.

Particular attention was given to studies reporting serial CMR examinations after acute myocarditis, with emphasis on temporal changes in tissue-characterization markers and their relationship with clinical outcomes. Studies were selected according to their relevance to the longitudinal evolution of CMR findings rather than according to a formal systematic-review protocol.

Contemporary international guidelines and consensus documents were prioritized to provide the clinical and conceptual framework, including the 2025 ESC Guidelines and the 2026 EHRA/HFA consensus statement.

Reference lists of relevant studies and reviews were also examined to identify additional longitudinal cohorts.

Given the narrative nature of this review, no formal risk-of-bias assessment or meta-analysis was performed.

## 3. What Are We Imaging in Myocarditis with CMR?

CMR provides a multiparametric assessment of suspected myocarditis by combining cardiac morphology and function with complementary markers of myocardial tissue composition. A comprehensive examination should therefore be considered as a sequence of acquisitions addressing different components of myocardial injury rather than as a single diagnostic test. Cine imaging defines the functional phenotype, T2-sensitive imaging and parametric mapping assess myocardial water content and tissue composition, ECV quantifies extracellular expansion, whereas LGE primarily depicts focal differences in gadolinium distribution. The integration of these findings, rather than any individual CMR abnormality, forms the basis for the non-invasive characterization of myocardial inflammation [4,23].

Importantly, the diagnostic performance of CMR is strongly influenced by the timing of imaging. Diagnostic accuracy is highest when CMR is performed early in the disease course, ideally within the first 2 weeks after symptom onset, when myocardial oedema and other inflammation-related tissue abnormalities are most likely to be detectable; as these abnormalities progressively resolve, the sensitivity of CMR for identifying the acute inflammatory phenotype may decrease [2,24].

**Figure 1:**
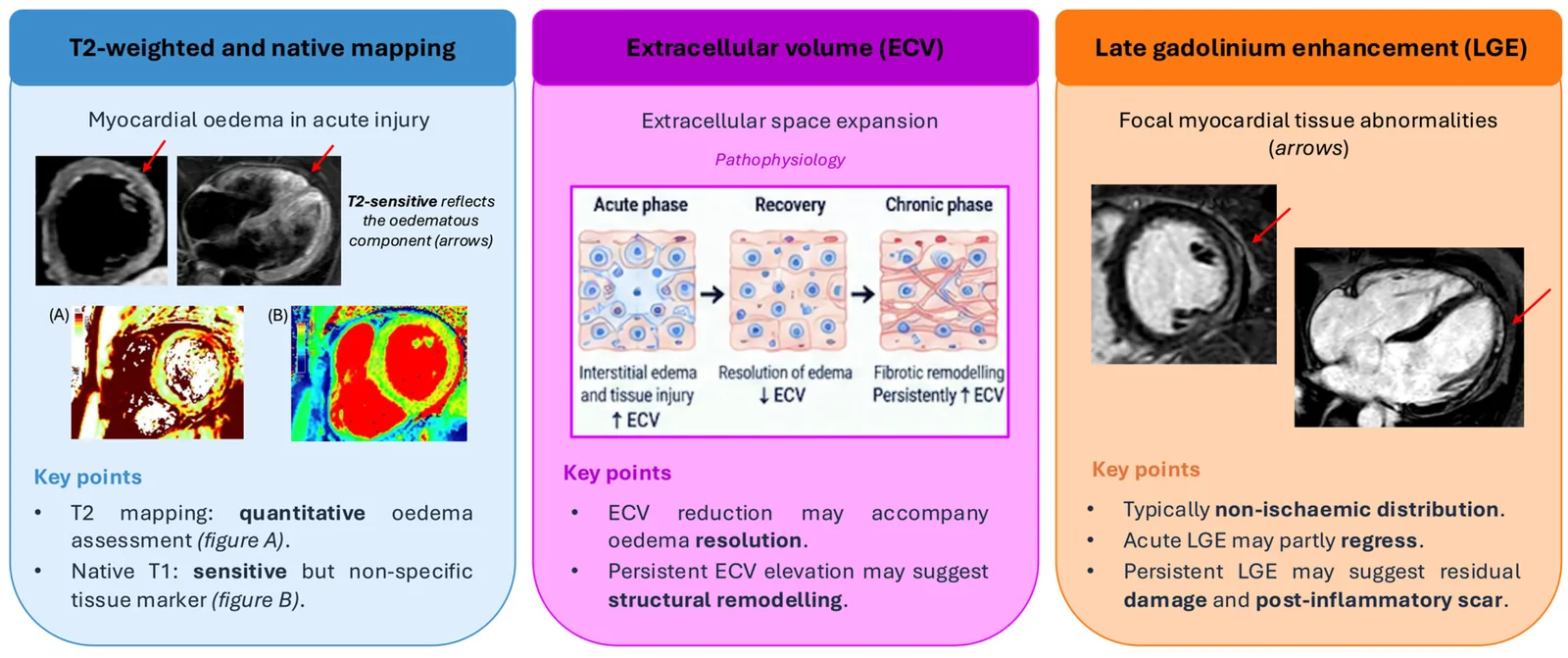
Complementary information provided by CMR tissue markers in myocarditis. **Left** panel (T2-weighted imaging and native mapping): T2-weighted imaging detects myocardial oedema associated with acute inflammation (arrows), while T2 mapping enables quantitative assessment of oedema and native T1 mapping provides a sensitive, although non-specific, marker of myocardial tissue abnormalities. **Middle** panel (extracellular volume, ECV): schematic representation of changes in the myocardial extracellular space throughout the disease course; ECV elevation during the acute phase reflects interstitial oedema and tissue injury; ECV may decrease with oedema resolution during recovery, whereas persistent elevation in the chronic phase may indicate fibrotic remodelling. **Right** panel (late gadolinium enhancement, LGE): representative CMR images showing focal myocardial tissue abnormalities with a typically non-ischaemic distribution (arrows); LGE may partially regress following the acute phase, whereas persistent enhancement may reflect residual myocardial damage and post-inflammatory scar. Abbreviations: CMR, cardiovascular magnetic resonance; ECV, extracellular volume; LGE, late gadolinium enhancement.

As illustrated in Figure 1, multiparametric CMR interrogates complementary components of myocardial injury, including myocardial water content and tissue composition, extracellular compartment expansion, and focal myocardial injury or residual LGE and potential structural remodelling, whose biological significance varies according to the timing of imaging and should therefore be interpreted in an integrated manner.

Importantly, CMR tissue markers provide complementary but not histologically specific information on myocardial injury. None can, when considered in isolation, determine the underlying biological substrate with complete specificity. Their interpretation therefore depends on the timing of imaging, the clinical context, and integration with the other CMR findings. These general limitations apply to the individual markers discussed below and are not reiterated in each subsection.

### 3.1. Cine Imaging: Morphology and Ventricular Function

A CMR examination generally begins with cine imaging, which provides a comprehensive assessment of cardiac morphology, ventricular volumes, and global and regional biventricular systolic function. In patients with myocarditis, ventricular function may range from completely preserved to severe global or regional dysfunction, depending on the extent and clinical phenotype of myocardial involvement. When present, regional wall motion abnormalities may show a non-coronary distribution, reflecting the heterogeneous and patchy nature of myocardial involvement rather than conforming to a single epicardial coronary territory. Cine imaging also allows assessment of myocardial wall thickness, which may transiently increase in the presence of substantial myocardial oedema, as well as associated findings such as pericardial effusion [2]. More specifically, acute myocarditis may result in an apparent hypertrophic or “pseudo-hypertrophic” phenotype. Histopathological studies have demonstrated that this phenomenon predominantly reflects interstitial myocardial oedema rather than true cardiomyocyte hypertrophy, with wall thickness decreasing during convalescence despite no significant change in cardiomyocyte diameter [25]. Consistently, serial CMR studies have demonstrated a reduction in LV mass during recovery from acute myocarditis in parallel with the resolution of myocardial oedema [26]. Recognition of this transient morphological phenotype is clinically relevant, as marked oedema-related wall thickening may mimic a hypertrophic phenotype during the acute phase; regression of wall thickness and myocardial mass together with resolution of oedema at follow-up supports its transient nature.

Importantly, however, preserved left ventricular ejection fraction does not exclude significant myocardial injury, particularly in patients with an infarct-like presentation [27]. This apparent dissociation can partly be explained by the complex transmural architecture of myocardial contraction. The subendocardial layer, despite accounting for less than approximately 20% of myocardial wall thickness, contains predominantly longitudinally oriented fibres and plays a major role in longitudinal left ventricular mechanics, whereas mid-wall and subepicardial fibres contribute importantly to circumferential shortening, wall thickening, and rotational mechanics [28]. Consequently, early or spatially limited impairment of one component of myocardial deformation may be partially compensated by preserved or enhanced mechanics in other myocardial layers, allowing global LVEF and visually assessed wall motion to remain apparently normal despite underlying myocardial injury [27,28]. Moreover, conventional cine imaging primarily evaluates changes in ventricular volumes, wall thickening, and macroscopic wall motion and does not directly characterize the complex three-dimensional and transmural deformation of myocardial fibres [27,29].

More sensitive measures of myocardial mechanics, including strain assessment by CMR feature tracking, tagging, or other deformation techniques, may therefore reveal subtle functional abnormalities that are not apparent from LVEF alone [27,28,29,30]. Functional assessment should consequently be considered complementary to, rather than a surrogate for, myocardial tissue characterization, since preserved global systolic function neither excludes myocardial injury nor necessarily implies complete myocardial recovery.

### 3.2. STIR Sequences and T2-Weighted Imaging: Acute Myocardial Injury

Once cardiac morphology and function have been evaluated, conventional T2-weighted imaging represents the first step in myocardial tissue characterization and is primarily aimed at detecting increased myocardial water content [4,31]. Acute myocardial oedema (AMO), defined as an increase in myocardial water content, is an early response to inflammatory activation and increased vascular permeability in acute myocardial injury. It initially reflects intracellular water accumulation (cytotoxic oedema), followed by expansion of the interstitial compartment (vasogenic oedema) [32,33,34]. Myocardial oedema prolongs T2 relaxation and consequently results in increased signal intensity on T2-weighted images, providing an imaging marker of increased myocardial water content [4,31,32].

Short-tau inversion recovery (STIR) sequences, typically acquired using a black-blood triple-inversion recovery technique, suppress the signal from flowing blood and fat while preserving T2-weighted contrast, thereby allowing areas of myocardial oedema to appear hyperintense relative to unaffected myocardium [31]. In acute myocarditis, increased T2-weighted signal may be regional or diffuse and commonly shows a non-ischaemic distribution. Typically, acute myocardial oedema predominantly involves the mid-myocardial or subepicardial layers and may affect different myocardial regions, with frequent involvement of the inferolateral wall [35]. Conventional T2-weighted imaging therefore provides a qualitative or semi-quantitative marker of myocardial oedema and represented the principal T2-based approach in the original Lake Louise Criteria [31].

Historically, myocardial oedema on T2-weighted imaging has been assessed either visually or semi-quantitatively using the T2 signal-intensity ratio, calculated by comparing myocardial signal intensity with that of skeletal muscle within the same image. A myocardial-to-skeletal-muscle signal-intensity ratio ≥ 2.0 was proposed as evidence of myocardial oedema in the original Lake Louise Criteria [31]. However, this approach has several important technical limitations. Image quality may be affected by respiratory and cardiac motion, incomplete blood suppression, surface-coil inhomogeneity, and low signal-to-noise ratio. Furthermore, skeletal muscle is not always an appropriate internal reference, particularly in systemic inflammatory disorders in which skeletal muscle may itself be involved. These limitations reduce the sensitivity of conventional T2-weighted imaging, particularly when myocardial involvement is mild or diffuse rather than focal [4,31].

These limitations have progressively favoured the use of quantitative T2 mapping, which provides direct pixel-wise measurements of myocardial T2 relaxation time rather than relying on relative signal intensity and is discussed in detail below [4,23]. Conventional T2-weighted imaging nevertheless remains useful as a qualitative assessment of the presence and spatial distribution of myocardial oedema.

### 3.3. Native T1 Mapping

Native T1 mapping provides a quantitative, contrast-free assessment of myocardial tissue composition. T1 represents the longitudinal relaxation time of myocardial tissue and is influenced by both intracellular and extracellular components. In normal myocardium, native T1 values reflect the composite signal arising from cardiomyocytes and the interstitial space, whereas pathological changes that increase tissue water content or expand the extracellular compartment generally result in prolongation of myocardial T1 relaxation times [4,23,36]. Native T1 values are field-strength-, sequence-, vendor-, and site-dependent; therefore, absolute measurements should always be interpreted against locally validated reference ranges rather than using universal cut-off values [23,36].

In acute myocarditis, native T1 is typically increased as a consequence of myocardial oedema, inflammation-related tissue injury, and expansion of the interstitial space. Unlike T2, which more closely reflects changes in myocardial water content, native T1 is sensitive to a broader spectrum of tissue abnormalities. Increased native T1 may therefore identify both focal and diffuse myocardial involvement and can remain abnormal even when conventional T2-weighted imaging is inconclusive [4,23,37]. Because native T1 mapping does not require administration of gadolinium-based contrast agents, it also provides tissue-characterization information when contrast administration is contraindicated or undesirable [4,23].

A major advantage of native T1 mapping is its quantitative and pixel-wise nature. Conventional LGE relies on differences in signal intensity between abnormal and relatively normal myocardium and may therefore be less sensitive to homogeneous or diffuse myocardial abnormalities. Native T1 mapping, by assigning an absolute T1 value to each voxel, can detect diffuse alterations in myocardial composition even in the absence of a visually apparent regional reference. This feature is particularly relevant in inflammatory myocardial disease, in which myocardial involvement may extend beyond areas showing visually detectable LGE [4,23,37].

This distinction becomes particularly important when T1 and T2 are considered together. Concomitant elevation of native T1 and T2 in the acute clinical setting is consistent with myocardial injury occurring in the presence of increased myocardial water content, whereas persistent native T1 elevation after normalization of T2 may reflect residual extracellular expansion or structural remodelling rather than ongoing oedema [4,15,37].

The temporal behaviour of native T1 may therefore provide additional information during follow-up. Native T1 values generally decrease as acute myocardial injury and oedema resolve, although their evolution may not parallel that of T2 because the two parameters reflect partially different tissue properties [37,38]. Persistent native T1 abnormalities after normalization of T2 may identify residual myocardial alterations and should be interpreted together with ECV and LGE to help characterize whether the imaging phenotype is evolving from an oedema-dominant acute injury toward a more stable pattern of structural remodelling [15,37,38]. Accordingly, the greatest value of native T1 in myocarditis lies not in its interpretation as an isolated biomarker, but in its integration within a multiparametric and longitudinal CMR assessment.

### 3.4. T2 Mapping

Unlike conventional T2-weighted imaging, which relies on relative differences in signal intensity between myocardium and reference tissues, T2 mapping generates a pixel-wise parametric map in which each voxel is assigned an absolute T2 relaxation time, expressed in milliseconds [4,23]. Different acquisition strategies are available, most based on T2-prepared balanced steady-state free-precession sequences acquired with different T2 preparation times, from which the myocardial T2 relaxation curve is derived [23].

The biological basis of T2 mapping relies on the close relationship between T2 relaxation and tissue water content. In acute myocardial injury, intracellular and interstitial water accumulation prolongs T2 relaxation, resulting in increased myocardial T2 values. In acute myocarditis, elevated T2 therefore provides quantitative evidence of increased myocardial water content and represents a T2-based criterion within the updated Lake Louise Criteria [4]. Compared with native T1, T2 is more closely related to tissue water content and is less influenced by chronic extracellular expansion and fibrosis, making it particularly useful for identifying the oedematous component of acute myocardial injury [4,23,37].

An important advantage of T2 mapping over conventional T2-weighted imaging is its ability to detect diffuse myocardial abnormalities. When myocardial oedema is widespread, conventional T2-weighted techniques may fail to demonstrate a clear regional difference because apparently normal myocardium may no longer provide an adequate internal reference. Similarly, calculation of the myocardial-to-skeletal-muscle signal-intensity ratio may be unreliable when skeletal muscle is affected by systemic inflammatory disease. By providing direct quantitative measurements of myocardial T2 without requiring comparison with skeletal muscle, T2 mapping partly overcomes these limitations and improves the detection of diffuse or subtle increases in myocardial water content [4,23].

Nevertheless, T2 mapping should not be regarded as an absolute or technique-independent measurement. Normal T2 values depend on magnetic field strength, pulse sequence, acquisition parameters, scanner platform, and post-processing methodology, and substantial differences may exist between centres. Consequently, T2 values should be interpreted using sequence- and site-specific reference ranges rather than universal numerical cut-offs [23]. Technical factors including motion, partial-volume effects, inadequate breath-holding, susceptibility artefacts and contamination from blood or adjacent tissues may also influence measured T2 values, particularly in the subendocardial and subepicardial regions [23].

The temporal evolution of T2 abnormalities is particularly relevant in myocarditis. Owing to its quantitative nature, T2 mapping allows changes in myocardial water content to be assessed serially over time. In uncomplicated acute myocarditis, myocardial T2 values generally decrease during follow-up, broadly paralleling resolution of myocardial oedema [19,20,38]. A progressive decline toward the institutional reference range is therefore consistent with resolution of the oedematous component of myocardial injury, whereas persistently or recurrently elevated T2 may indicate delayed resolution of myocardial oedema and, in the appropriate clinical context, raise suspicion of ongoing inflammatory activity [19,20,38]. However, the timing of T2 normalization is variable, and no universally validated T2 threshold distinguishes active from healed myocarditis.

The relationship between T2 and the other tissue markers becomes particularly informative during follow-up. Concomitant elevation of T2 and native T1 in the appropriate clinical setting supports an oedema-rich acute myocardial injury phenotype, whereas normalization of T2 with persistence of T1 abnormalities or LGE suggests a transition away from predominantly oedematous injury toward residual extracellular or structural abnormalities [15,37,38]. Thus, rather than providing a binary marker of active versus inactive myocarditis, T2 mapping contributes to a multiparametric characterization of the myocardial phenotype and, particularly when assessed serially, may help define the transition from acute injury toward myocardial healing.

The prognostic significance of myocardial oedema is less straightforward. Rather than representing an adverse prognostic marker per se, acute myocardial oedema reflects an active and potentially reversible component of myocardial injury [39,40]. However, neither the presence nor the extent of myocardial oedema independently predicted long-term outcome, for which impaired baseline LVEF remained the main independent CMR predictor [40]. Greater global myocardial inflammation has similarly been associated with a higher likelihood of subsequent recovery of LVEF [41]. These observations suggest that the prognostic meaning of an oedema-dominant acute phenotype may differ from that of persistent structural myocardial injury, although the mechanisms underlying these associations remain incompletely understood [39,40,41]. Accordingly, myocardial oedema should not be considered as an isolated prognostic marker but interpreted together with ventricular function, LGE burden and distribution, timing of CMR, arrhythmic presentation, and the overall clinical phenotype [2,42,43].

### 3.5. Contrast Agent Administration and Extracellular Volume (ECV)

Following administration of a gadolinium-based extracellular contrast agent, post-contrast T1 mapping can be combined with native T1 measurements and blood haematocrit to calculate the myocardial ECV. ECV represents the proportion of myocardial tissue occupied by the extracellular compartment and is derived from the relative change in myocardial and blood T1 relaxation rates before and after contrast administration, corrected for the patient’s haematocrit [23,36]. Because gadolinium distributes within the extracellular space but does not cross intact cell membranes, ECV provides a quantitative estimate of extracellular space expansion and, unlike LGE, does not rely primarily on the presence of a relatively normal myocardial reference region. Post-contrast T1 mapping is generally performed after sufficient contrast equilibration, typically approximately 10–30 min after contrast administration, although acquisition protocols should follow sequence- and site-specific recommendations [23].

In acute myocarditis, ECV may increase because of interstitial oedema, tissue injury, and extracellular matrix expansion [15,23]. Increased ECV during the acute phase should therefore not be interpreted as synonymous with myocardial fibrosis, because potentially reversible inflammatory oedema and irreversible structural changes may both contribute to extracellular expansion [15,23].

This distinction is particularly important when ECV is interpreted together with native T1 and T2 mapping. Whereas T2 is more closely related to myocardial water content, native T1 is influenced by changes in both intracellular and extracellular tissue composition, while ECV more specifically quantifies expansion of the extracellular compartment. In the acute setting, concomitant increases in T2, native T1, and ECV may therefore characterize an oedema-rich inflammatory injury phenotype. Conversely, normalization of T2 accompanied by persistent ECV expansion may suggest that the extracellular abnormality is no longer predominantly driven by oedema and may increasingly reflect residual structural remodelling [15,37].

The longitudinal interpretation of ECV is therefore attractive but complex. In principle, a reduction in ECV occurring in parallel with normalization of T2 may reflect resolution of inflammatory oedema and contraction of the previously expanded extracellular compartment, whereas persistent ECV elevation may be consistent with residual extracellular matrix expansion or structural remodelling, potentially including a fibrotic component [14,15]. This temporal framework may be particularly useful in myocarditis because the biological meaning of an elevated ECV is likely to change throughout the disease course: extracellular expansion during the acute phase may contain a substantial reversible component, while persistent abnormalities at later follow-up are more likely to represent structural myocardial alteration.

However, evidence supporting the prognostic significance of serial ECV changes in myocarditis remains limited, and validated thresholds distinguishing reversible inflammatory extracellular expansion from irreversible fibrosis are currently lacking. Indeed, the relationship between ECV and histological fibrosis may itself be influenced by concomitant myocardial inflammation, further limiting the specificity of ECV as a non-invasive surrogate of fibrosis during the inflammatory phase [37].

### 3.6. LGE: Myocardial Injury and Residual Tissue Abnormalities

LGE imaging is generally performed approximately 10–15 min after administration of a gadolinium-based contrast agent, according to protocol, using inversion-recovery sequences in which the signal of normal myocardium is nulled. Regions with relatively greater gadolinium retention appear hyperintense, allowing the detection and spatial characterization of focal myocardial injury [23,31]. Unlike ECV, which provides a quantitative estimate of extracellular space expansion, LGE primarily provides spatial information on the presence, distribution, and extent of regional myocardial abnormalities, making the anatomical pattern of enhancement an integral component of its interpretation.

In acute myocarditis, LGE characteristically follows a non-ischaemic distribution, most commonly involving the subepicardial or mid-myocardial layers, with a frequent predilection for the inferolateral wall [4,31,44]. This pattern differs from the subendocardial or transmural enhancement conforming to a coronary vascular territory that characterizes ischaemic myocardial injury. Nevertheless, considerable heterogeneity exists, and LGE may involve other myocardial regions according to the underlying aetiology and clinical phenotype. In particular, mid-wall septal involvement has been repeatedly described and appears to carry important prognostic implications [42,43,45,46].

Importantly, LGE should not be regarded as synonymous with myocardial fibrosis, particularly when CMR is performed during the acute phase. Acute inflammatory injury may produce enhancement that subsequently decreases or disappears as the acute process resolves. Serial CMR studies have demonstrated substantial reductions in LGE burden during follow-up, indicating that at least part of the enhancement observed at presentation does not represent irreversible replacement fibrosis [19,20]. Consequently, the biological meaning of LGE is inherently dependent on the timing of imaging.

This temporal dependence becomes particularly relevant after resolution of myocardial oedema. Persistent LGE after normalization of T2 may reflect residual myocardial tissue abnormalities potentially consistent with fibrosis or structural remodelling. However, LGE cannot independently establish the presence of histologically confirmed replacement fibrosis, and normalization of oedema-sensitive markers does not exclude residual low-grade inflammatory activity. Therefore, these findings should be interpreted in the context of the clinical course and other diagnostic information [19,20]. Conversely, reduction or disappearance of LGE during follow-up supports the concept that acute enhancement may contain a reversible component and highlights the limitations of using a single acute CMR examination to define the definitive scar burden.

Beyond its diagnostic role, LGE provides important prognostic and arrhythmic information. Its prognostic significance appears to depend not only on its presence or extent but also on its anatomical distribution [42,43,45,46,47]. Septal, particularly mid-wall septal, LGE has been associated with a higher risk of adverse cardiovascular events and incomplete recovery, including among patients with preserved LVEF [42,45,46]. Meta-analytic evidence further supports an association between LGE and adverse outcomes in myocarditis, although differences in patient populations, timing of CMR, and definitions of clinical endpoints should be considered when interpreting these data [43,48].

The prognostic significance of LGE may itself evolve over time. Acute LGE burden may be less informative than residual LGE assessed after myocardial oedema has resolved, when enhancement is more likely to reflect a stable structural substrate [19,20]. In this setting, persistent LGE, as a potential marker of residual structural substrate, may provide an anatomical substrate for ventricular arrhythmias even after normalization of ventricular function and resolution of other markers of acute inflammation. Thus, the clinically relevant post-myocarditis LGE phenotype should be considered multidimensional and time-dependent, incorporating presence, extent, anatomical distribution, relationship with oedema, and temporal evolution, rather than simply classifying LGE as present or absent.

### 3.7. Pattern Matters

Beyond its presence and extent, the anatomical distribution of LGE is a fundamental component of CMR interpretation. In myocarditis, LGE typically follows a non-ischemic pattern, most commonly involving the subepicardial or mid-myocardial layers, with a predilection for the basal-to-mid inferolateral wall [4,31,44]. Conversely, myocardial infarction characteristically produces LGE beginning in the subendocardium, reflecting the greater vulnerability of this layer to ischemia, with variable extension toward the epicardium according to the severity and duration of coronary occlusion. Thus, an ischemic pattern is defined not simply by the transmural extent of enhancement, but by involvement of the subendocardium in a distribution corresponding to the perfusion territory of an epicardial coronary artery [4,31].

Accordingly, the distinction between ischaemic and non-ischaemic injury is generally straightforward when LGE is confined to the subepicardial or mid-myocardial layers. However, inflammatory injury may occasionally extend transmurally, including in patients with infarct-like myocarditis [49]. Transmurality should therefore not be considered synonymous with an ischaemic origin. In this setting, the overall spatial distribution of enhancement and its relationship to coronary territories are more informative than transmurality per se: subendocardial-to-transmural enhancement conforming to a coronary vascular territory strongly favours an ischaemic mechanism, whereas patchy or multifocal transmural enhancement extending beyond a single coronary territory may remain compatible with inflammatory myocardial injury [4,31,49,50].

In patients with an infarct-like presentation, coronary anatomy and the overall clinical context remain essential when LGE is atypical or transmural, since co-localization of LGE with myocardial oedema does not by itself distinguish inflammatory from ischaemic injury [4,31].

Finally, pattern also carries prognostic information within myocarditis itself. The typical inferolateral subepicardial pattern should be distinguished from mid-wall anteroseptal involvement, which has been associated with a less favorable outcome even in patients with preserved LVEF. In the ITAMY study, anteroseptal mid-wall LGE was independently associated with adverse outcomes compared with other LGE patterns, emphasizing that the clinical significance of LGE depends not only on how much enhancement is present, but also on where within the myocardium it is located [42].

### 3.8. Technical Standardization and Reproducibility in Serial CMR

The interpretation of serial CMR requires particular attention to measurement reproducibility, because an apparent change in a quantitative tissue marker may reflect technical variability rather than true biological evolution. This consideration is especially important for native T1, T2, and ECV, for which relatively small numerical differences between examinations may otherwise be interpreted as resolution or progression of myocardial abnormalities. Whenever possible, longitudinal examinations should therefore be performed on the same scanner platform and at the same magnetic field strength, using identical pulse sequences, acquisition parameters, reconstruction methods, and post-processing procedures [23,51]. Changes in scanner hardware or software, sequence implementation, reconstruction algorithms, or post-processing software should be documented and considered when serial measurements are compared [23].

Consistency of image acquisition and analysis is equally important. Serial mapping should ideally use comparable cardiac acquisition phases, slice positions, motion-correction approaches, and myocardial segmentation and region-of-interest strategies, as differences in these factors may alter measured relaxation times independently of biological change [23,52]. Particular care is required at myocardial borders to minimize partial-volume contamination from blood pool or epicardial tissues. Where scanner or sequence changes are unavoidable, direct comparison of absolute values should be approached cautiously and should rely on appropriately validated reference ranges and knowledge of the precision and reproducibility of the specific mapping technique [52].

ECV introduces additional sources of longitudinal variability because its calculation depends on native and post-contrast myocardial and blood T1 values as well as haematocrit. Serial ECV assessment should therefore use comparable contrast-agent type and dose, post-contrast acquisition timing, and T1-mapping methodology, with a contemporaneous haematocrit measurement whenever possible. Similarly, longitudinal assessment of LGE should account for contrast dose and timing, acquisition and reconstruction technique, and the method used to define and quantify enhanced myocardium, since differences in thresholding or segmentation may influence apparent changes in LGE burden [51,52].

These technical considerations are particularly relevant when serial CMR is used to classify an individual patient as showing resolving, persistent, or recurrent abnormalities. Changes that are small relative to the known measurement variability of the technique should not automatically be interpreted as biological improvement or deterioration [53]. Greater confidence can be assigned to longitudinal change when it is concordant across multiple CMR parameters, exceeds expected technical variability, and is supported by the clinical, biomarker, functional, or rhythm trajectory. Thus, standardization of acquisition and post-processing is a prerequisite for interpreting quantitative serial CMR as a marker of disease evolution rather than measurement variability [53,54].

Importantly, small numerical differences between serial examinations should not automatically be interpreted as biological improvement or deterioration. The magnitude of a meaningful intraindividual change depends on the precision and scan–rescan reproducibility of the specific acquisition and analysis approach. Greater confidence can be assigned to longitudinal change when it exceeds the expected measurement variability of the technique, is observed under comparable acquisition and processing conditions, and is concordant across multiple CMR parameters and with the clinical, biomarker, functional, or rhythm trajectory.

The principal technical requirements for reliable comparison of serial CMR examinations are summarized in Table 2.

## 4. Myocarditis as an Imaging Trajectory

### 4.1. From a Diagnostic Event to a Dynamic Disease

Myocarditis should not be regarded as a single acute event, as the different biological components of the disease may evolve and recover at different rates. In most patients, the initial inflammatory response is followed by myocardial injury and a gradual resolution of inflammation, eventually leading to tissue resolution after the acute phase, with or without residual LGE and potential structural remodelling. In some cases, however, inflammation may persist or recur, resulting in ongoing myocardial damage and, ultimately, progression towards an inflammatory cardiomyopathy. These phases are not clearly separated and may overlap considerably between individual patients [1,55].

Clinical, biochemical, functional, and tissue recovery do not necessarily occur in parallel. Symptoms and cardiac biomarkers may normalize before complete recovery of ventricular function or resolution of myocardial abnormalities. Similarly, normalization of left ventricular ejection fraction does not necessarily indicate complete myocardial healing. Thus, patients who are asymptomatic and have normal troponin levels and preserved ventricular function may still show evidence of myocardial edema or residual LGE [2].

This temporal dissociation is particularly relevant when interpreting imaging findings during follow-up. Cardiovascular imaging, and CMR in particular, provides information that cannot be inferred from clinical or biochemical recovery alone, allowing assessment of residual myocardial inflammation and structural damage throughout the course of the disease.

### 4.2. CMR as the Cornerstone of Non-Invasive Tissue Characterization

CMR has become central to the evaluation of suspected myocarditis because it simultaneously provides ventricular morphology, biventricular function, myocardial tissue characterization, and assessment of associated pericardial involvement [3].

As outlined above, the updated Lake Louise Criteria provide a multiparametric framework integrating T2-based evidence of increased myocardial water content with T1-based evidence of myocardial injury [2,4]. These criteria remain the reference standard for CMR assessment in the acute phase. However, they were developed to identify CMR findings supporting acute myocardial inflammation rather than to characterize abnormalities persisting during follow-up. In this setting, distinguishing imaging findings compatible with ongoing inflammatory activity from residual post-inflammatory structural abnormalities remains a key challenge. Follow-up CMR therefore serves a different purpose, helping to distinguish persistent inflammatory alterations from residual structural damage and to better define the post-inflammatory myocardial substrate.

The diagnostic strength of CMR in myocarditis lies in the integration of complementary imaging findings rather than in any single abnormal parameter. Individual CMR markers interrogate different components of myocardial injury and may show limited diagnostic performance when considered in isolation; conversely, the concordance of multiple abnormalities increases the confidence that the observed imaging phenotype reflects clinically relevant myocardial injury. In a biopsy-referenced cohort of 132 patients with suspected myocarditis, Lurz et al. demonstrated that a multiparametric approach based on the conventional Lake Louise Criteria provided better diagnostic performance than individual CMR parameters, particularly in acute presentations. Using the presence of at least two of three conventional criteria, CMR showed a sensitivity of 76%, specificity of 54%, and diagnostic accuracy of 68% in the overall population, increasing to 81%, 71%, and 79%, respectively, in patients with acute myocarditis [24]. Importantly, however, diagnostic performance was substantially lower in patients with chronic presentations, emphasizing that the value of multiparametric CMR is strongly dependent on disease stage and timing of imaging.

The influence of the clinical phenotype was further demonstrated by Francone et al. in patients with biopsy-proven acute myocarditis. CMR sensitivity varied according to clinical presentation and the extent and type of myocardial cell injury, highlighting that the probability of detecting characteristic CMR abnormalities is not uniform across the spectrum of myocarditis [56]. Thus, even a comprehensive CMR examination may be less sensitive in phenotypes in which myocardial injury is less extensive or produces less conspicuous tissue abnormalities, reinforcing the need to interpret negative or equivocal CMR findings according to the clinical presentation and timing of the examination.

The introduction of quantitative tissue characterization has further expanded this multiparametric approach. Radunski et al. demonstrated that quantitative T1, T2, and ECV measurements provide additional diagnostic information in patients with severe myocarditis, with selected combinations of quantitative and conventional CMR parameters improving diagnostic accuracy compared with conventional criteria alone [57].

Accordingly, the diagnostic contribution of CMR should not be viewed as simple accumulation of abnormal findings, but rather as the concordant integration of complementary markers of myocardial water content, tissue composition, extracellular expansion, and focal injury. The greater the concordance of these findings within an appropriate clinical setting, the stronger the imaging evidence supporting myocarditis; nevertheless, CMR does not provide direct histological or aetiological characterization, and its diagnostic performance remains influenced by clinical phenotype, disease severity, and—critically—the timing of imaging.

## 5. Serial CMR and the Transition from Inflammation to Post-Acute Phase

Longitudinal CMR studies provide some of the most compelling evidence that the imaging phenotype of myocarditis changes substantially after the acute presentation.

However, the available evidence is derived from heterogeneous cohorts that differ considerably in sample size, clinical phenotype, timing of follow-up, CMR acquisition protocols, availability of parametric mapping, and methods used for LGE assessment. These differences should be considered when interpreting the relative strength and generalizability of individual studies. The principal longitudinal CMR cohorts informing the temporal framework discussed in this review are summarized in Table 3.

### 5.1. The Six-Month Perspective

Aquaro et al. evaluated 187 patients with acute myocarditis who underwent CMR at presentation and at 6-month follow-up. Myocardial edema resolved in most patients, whereas LGE showed a more variable course, persisting in the majority but decreasing or completely disappearing in a substantial proportion of cases. These findings indicate that LGE detected during the acute phase does not necessarily represent irreversible myocardial fibrosis, as part of the initial enhancement may regress with resolution of edema and acute myocardial injury. Importantly, persistent LGE after edema resolution was associated with a worse prognosis, particularly when a mid-wall septal pattern was present [20].

Accordingly, the progressive reduction in LGE burden observed during follow-up may reflect resolution of the reversible components contributing to acute enhancement and/or progressive remodelling of the residual myocardial injury, a phenomenon commonly referred to as “scar shrinkage”. Importantly, changes in LGE extent should not be interpreted as direct evidence of histological regression of fibrosis, as CMR provides an imaging surrogate of tissue injury and direct longitudinal validation against histopathology is lacking. Accordingly, even complete disappearance of visually detectable LGE does not necessarily imply complete histological restitution, since a small residual scar may theoretically shrink below the spatial resolution of CMR. Conversely, LGE persisting after resolution of oedema is more likely to reflect the residual post-inflammatory structural substrate and may therefore provide a more representative estimate of residual post-inflammatory scar burden [20].

These findings emphasize the importance of considering the timing of CMR when interpreting LGE in myocarditis. While acute-phase LGE reflects myocardial injury in the setting of acute inflammation, its persistence after oedema resolution is more consistent with residual structural myocardial abnormality, although it does not by itself establish mature replacement fibrosis histologically. Thus, the prognostic meaning of LGE may become clearer once the inflammatory edema has resolved.

### 5.2. The Potential Role of Three-Month Reassessment

Observational data have explored whether CMR reassessment at approximately three months may provide clinically relevant information in selected patients with uncomplicated acute myocarditis. This time point should not be regarded as defining a universally mature or definitive post-myocarditis scar, but rather as a potentially informative stage within a continuing process of myocardial healing.

Pieroni and colleagues performed CMR at presentation, three months, and twelve months in 33 patients with uncomplicated acute myocarditis, predominantly young men with an infarct-like presentation. Between baseline and three months, myocardial oedema markedly decreased, LGE burden declined, LVEF improved, and left ventricular mass decreased. Oedema persisted in a minority of patients at three months and subsequently resolved, whereas among patients in whom oedema had resolved, relatively little additional change in LGE was observed between three and twelve months [19]. These findings suggest that, in selected patients with an uncomplicated clinical course, a three-month examination may provide useful information on the transition from acute injury toward a more stable residual imaging substrate.

However, these observations should be interpreted as hypothesis-generating rather than as evidence establishing three months as the optimal or definitive follow-up time point. The study included only 33 patients, had limited representation of severe or non-infarct-like myocarditis, and the reported association between follow-up LGE burden and persistent ventricular arrhythmias was based on a small number of patients with persistent arrhythmias. Accordingly, the findings should not be extrapolated to the broader spectrum of myocarditis. Rather, they support the potential value of three-month CMR in selected uncomplicated cases, particularly when assessment of oedema resolution and evolution of LGE may inform subsequent clinical evaluation [19].

Pieroni and colleagues performed CMR at presentation, three months, and twelve months in patients with uncomplicated acute myocarditis. Between baseline and three months, edema markedly decreased, LGE burden declined, LVEF improved, and left ventricular mass decreased. Edema persisted at three months in a minority of patients and subsequently resolved. Importantly, among patients in whom edema had resolved, relatively little additional change in LGE was observed between three and twelve months. Over long-term follow-up, the extent of LGE measured at three and twelve months was associated with persistent ventricular arrhythmias, whereas acute LGE burden showed a weaker relationship [19].

Although this was a relatively small study and its findings should not be generalized to all forms of myocarditis, it raises the hypothesis that CMR performed after oedema resolution may provide prognostically relevant information on residual myocardial abnormalities that is not apparent from acute-phase CMR. Persistent LGE may identify a myocardial substrate associated with subsequent ventricular arrhythmias; however, LGE is an imaging marker of myocardial tissue abnormality and should not be interpreted as a direct electrophysiological measure of a re-entrant arrhythmogenic substrate [37,58].

Whether similar temporal patterns apply to patients with fulminant, recurrent, arrhythmic, or specific aetiological forms of myocarditis remains uncertain and requires dedicated investigation.

### 5.3. Evolution of Arrhythmic Risk

One of the most clinically important consequences of myocarditis is the development of ventricular arrhythmias. Arrhythmic risk is dynamic and multifactorial and should not be interpreted as a simple linear transition from inflammation-mediated electrical instability during the acute phase to scar-mediated re-entry after myocardial healing. Active inflammation, evolving fibrosis, and established scar may coexist, and their relative contribution to arrhythmogenesis may change over time. Accordingly, arrhythmic risk depends not only on disease phase but also on the presenting arrhythmia, ventricular function, myocardial scar burden and topology, genetic background, and other clinical risk markers [2,3,59,60].

The 2026 EHRA/HFA clinical consensus statement provides a phase-aware framework for this evolving arrhythmic substrate, distinguishing hot, hot-to-cold, and cold phases [3]. During the hot phase, myocardial oedema, inflammation, and myocyte injury may promote electrical instability by affecting ion-channel function, calcium handling, and myocardial conduction, and polymorphic or pleomorphic ventricular arrhythmias may occur. As inflammation subsides, residual inflammatory activity may coexist with early reparative changes and developing fibrosis, creating a heterogeneous and potentially arrhythmogenic substrate. The hot-to-cold transition is therefore particularly relevant, as inflammation-related electrical instability may coexist with progressively developing re-entrant mechanisms. In the cold phase, residual replacement fibrosis becomes increasingly important, providing areas of slow conduction that may facilitate scar-related re-entry and sustained monomorphic ventricular tachycardia. Importantly, however, these phases should be regarded as predominant and potentially overlapping biological and electrophysiological states rather than as strictly sequential categories [2,3,59,60].

These mechanisms represent a conceptual pathophysiological framework rather than mechanisms directly demonstrated by serial CMR in individual patients. In particular, changes in oedema-sensitive markers and LGE over time cannot by themselves establish a transition from inflammation-mediated electrical instability to scar-mediated re-entry.

A wide spectrum of arrhythmias may occur throughout the course of myocarditis, and the arrhythmic presentation remains less well characterized than other clinical phenotypes. Polymorphic and irregular ventricular arrhythmias may occur more frequently in the presence of active inflammation, whereas monomorphic and regular ventricular arrhythmias are more commonly associated with a more organized arrhythmogenic substrate; however, substantial overlap exists, and arrhythmia morphology should not be regarded as a specific marker of disease phase or underlying mechanism. Moreover, myocarditis itself may remain unrecognized in patients presenting primarily with arrhythmias, making available data difficult to interpret. For these reasons, arrhythmic myocarditis represents a distinct clinical scenario that requires specific considerations in terms of diagnosis, risk stratification, and management [2,3,59,60].

Contemporary arrhythmic risk stratification should therefore integrate multiple dimensions rather than rely on disease phase or left ventricular ejection fraction alone. The 2026 EHRA/HFA consensus emphasizes a multiparametric framework incorporating the presenting and subsequent arrhythmic phenotype, CMR-defined LGE burden and topology, ventricular systolic function, unexplained syncope, electrophysiological findings, and genetic background [3]. NSVT, extensive LGE, unexplained syncope, positive electrophysiological testing, and reduced systolic function (LVEF <50%) are recognized risk markers for sudden cardiac death, while specific histological substrates and cardiomyopathy-associated genotypes may provide additional risk information [2,3]. Importantly, these features may coexist and interact rather than identifying mutually exclusive mechanisms of arrhythmogenesis. CMR contributes to this multidimensional assessment by characterizing both inflammation-related myocardial abnormalities and residual structural substrate, while LGE burden and distribution may provide prognostic information beyond ventricular function alone [2,3,42,61].

Genetic background represents an additional component of this framework. Pathogenic or likely pathogenic variants in cardiomyopathy-associated genes may predispose to ventricular arrhythmias, fibrotic remodelling, recurrent inflammatory episodes, and myocarditis-like presentations, suggesting that an apparent episode of myocarditis may in selected patients represent the inflammatory manifestation of an underlying genetically determined cardiomyopathic substrate. Genetic findings should not be interpreted as isolated determinants of risk, but integrated with family history, arrhythmic phenotype, ventricular function, CMR-defined myocardial substrate, and the clinical course [3].

Advances in cardiac imaging, particularly CMR, have improved the detection and characterization of myocardial inflammation and fibrosis while providing valuable information for arrhythmic risk stratification [4,42,61]. CMR may therefore provide imaging correlates across a dynamic and potentially overlapping spectrum ranging from inflammation-dominant electrical instability to heterogeneous healing and residual scar-related arrhythmogenic substrate. Repeat CMR after the acute phase may be considered in selected patients, particularly in those presenting with arrhythmias, persistent symptoms, impaired ventricular function, or extensive LGE at baseline. However, no universally validated quantitative LGE threshold currently guides ICD implantation. LGE findings should therefore not be interpreted in isolation for device decisions but considered in the broader context of disease phase, ventricular function, LGE burden and topology, presenting and subsequent arrhythmic phenotype, unexplained syncope, genetic background, and the overall clinical trajectory [1,2,3,62]. In this context, repeated reassessment is particularly important because the relative contribution of inflammation, evolving fibrosis, and established scar may change over time.

More broadly, these considerations support repeated multimodal risk assessment rather than classification based on a single examination or time point. Serial evaluation should integrate the clinical course, ECG and rhythm monitoring, ventricular function, CMR evidence of residual or evolving inflammation and scar, and, when clinically appropriate, genetic and electrophysiological information [2,3]. This is particularly relevant during the hot-to-cold transition, which should not simply be regarded as a period of declining arrhythmic risk, since residual inflammatory activity and an evolving fibrotic substrate may coexist and reshape arrhythmic vulnerability over time.

Future risk stratification may move beyond the simple assessment of LGE presence or extent towards a more detailed characterization of scar architecture. Features such as scar heterogeneity, border-zone characteristics, spatial distribution, and transmural involvement may provide additional information on the arrhythmogenic substrate. Integration of these imaging findings with electroanatomical mapping may further improve substrate characterization, strengthening the role of combined CMR and electrophysiological assessment in patients with previous myocarditis.

### 5.4. When Should Imaging Be Repeated?

The value of repeat imaging after myocarditis is increasingly recognized, although the timing of follow-up should likely be tailored to the clinical course rather than based on a fixed schedule. The 2025 ESC Guidelines recommend follow-up in all patients, combining clinical assessment with ECG, biomarkers, rhythm monitoring, echocardiography, and CMR according to disease severity and subsequent evolution [2].

Accordingly, the 2025 ESC Guidelines recommend structured follow-up in all patients with myocarditis, including CMR performed at least within the first 6 months after the index event (Class I, Level of Evidence C). Follow-up CMR contributes to the assessment of disease remission or persistence, characterization of residual myocardial abnormalities, and longitudinal risk stratification, thereby supporting individualized clinical management. Importantly, integration of CMR findings with symptoms, biomarkers, ventricular function, and rhythm assessment also contributes to determining the appropriate timing for a personalized return to work and physical activity [2]. CMR performed during the first months after the index event can provide a more complete picture of myocardial recovery by assessing whether oedema has resolved and ventricular function has recovered, while also documenting changes in LGE and the extent of residual scar. Persistent abnormalities may raise concern for ongoing myocardial injury and, when interpreted alongside the clinical course, may help determine whether further investigation is warranted and whether progression towards normal physical activity can be considered [1,20].

Available evidence suggests that CMR reassessment at approximately three and six months may provide complementary information, although these time points address different clinical questions and should not be considered universally interchangeable or optimal for all patients.

Aquaro et al., in a multicentre study including patients from the ITAMY cohort, demonstrated that CMR findings continue to evolve after the acute phase, with substantial changes in both myocardial oedema and LGE over the first six months. During the acute phase, LGE should not necessarily be interpreted as established fibrosis, as it may partly reflect reversible myocardial injury and can decrease or even disappear during follow-up. In contrast, LGE that persists at 6 months after myocardial oedema has resolved is more likely to represent residual fibrosis and has been associated with a less favourable prognosis, particularly when involving the mid-wall septal region. Persistent oedema, on the other hand, may indicate that the healing process is still ongoing and that further recovery remains possible [20].

Pieroni et al. suggested that in uncomplicated acute myocarditis, much of the edema resolution and LGE stabilization may already occur by three months [19].

These findings are not necessarily conflicting, as the two studies provide complementary information derived from different patient populations and follow-up protocols. Three-month reassessment may help characterize early oedema resolution and LGE evolution in selected uncomplicated cases, whereas evaluation at six months may provide additional information on subsequent tissue changes and the prognostic significance of persistent abnormalities.

Taken together, these observational studies should not be interpreted as defining a universal optimal time point for repeat CMR. Rather, they suggest that reassessment at approximately three months may be informative in selected uncomplicated cases, whereas evaluation later within the first six months may better capture continued tissue evolution in patients with persistent abnormalities or a more complex clinical course. Thus, the timing of repeat CMR within the guideline-recommended follow-up framework should be individualized according to the clinical phenotype, the course of recovery, and the specific question the examination is intended to address [2,19,20].

Earlier repeat CMR may be considered when the clinical course remains uncertain or recovery is incomplete. Persistent or recurrent symptoms, ongoing biomarker elevation, ventricular dysfunction, or clinically relevant arrhythmias may raise concern for unresolved myocardial injury and justify reassessment before the usual follow-up interval. An earlier examination may also be useful when uncertainty about persistent inflammatory activity could influence subsequent diagnostic or management decisions [2,3].

This time point may therefore help distinguish ongoing recovery from a more stable, scar-dominant pattern and, together with clinical and rhythm assessment, contribute to decisions regarding return to more intensive physical activity [19,20,63,64].

Further CMR beyond this period may be appropriate when myocardial abnormalities have not resolved or when the subsequent clinical course raises new concerns. Persistent oedema, progressive ventricular remodelling, or substantial residual scar, particularly when accompanied by ventricular arrhythmias, may justify continued imaging surveillance and consideration of an underlying inflammatory cardiomyopathy. In these patients, the timing of additional CMR should be individualized according to the evolution of both imaging and clinical findings [2,19].

Ultimately, rather than relying on a fixed follow-up interval, the timing of repeat CMR should be tailored to the individual clinical course and to the specific information sought from the examination.

## 6. Longitudinal CMR: From Active Inflammation to Post-Acute Phase

### 6.1. Temporal Changes in Tissue Markers

Serial CMR allows the relative evolution of oedema-sensitive and injury-related tissue markers to be assessed over time. In general, normalization of T2-sensitive abnormalities is consistent with resolution of the oedematous component of myocardial injury, whereas persistent T1/ECV abnormalities or LGE after T2 normalization may reflect residual extracellular or structural changes. Conversely, persistent or recurrent abnormalities across both T2- and T1-based markers may raise suspicion of delayed resolution or ongoing myocardial injury. These combinations should not be regarded as validated biological phenotypes but provide the basis for the longitudinal CMR patterns proposed below [2,4,15,19,20].

### 6.2. A “Mismatch” Between Imaging and Biology

Serial CMR may reveal a “mismatch” between imaging findings and the biological course of myocarditis, as clinical recovery, resolution of myocardial oedema, normalization of ventricular function, and regression of LGE do not necessarily occur in parallel. Moreover, the significance of individual CMR abnormalities may change over time. CMR findings should therefore be interpreted according to their temporal evolution and in the context of the overall clinical course [2,18,19,20].

Importantly, throughout this framework, terms describing inflammatory activity refer to CMR findings compatible with an inflammatory process rather than direct evidence of biologically or histologically active inflammation. None of the individual CMR markers—including T1, T2, ECV, or LGE—can independently establish the presence of ongoing myocardial inflammation. Conversely, normalization of oedema-sensitive markers does not establish complete histological resolution of inflammation. Because serial histological validation is rarely available, the biological substrates underlying longitudinal CMR changes remain incompletely defined.

Based on the temporal evolution of CMR abnormalities reported across longitudinal studies, different patterns of myocardial recovery may be recognized during follow-up. Given the heterogeneity and variable size of the available cohorts, these patterns have not been formally validated as distinct imaging phenotypes and should instead be regarded as a conceptual synthesis of the currently available longitudinal evidence. Nevertheless, they may provide a useful conceptual framework for interpreting serial CMR findings, ranging from near-complete imaging recovery to resolving or persistent oedema/injury-pattern abnormalities and post-acute myocardial injury with a residual LGE and potential structural remodelling.

**Figure 2:**
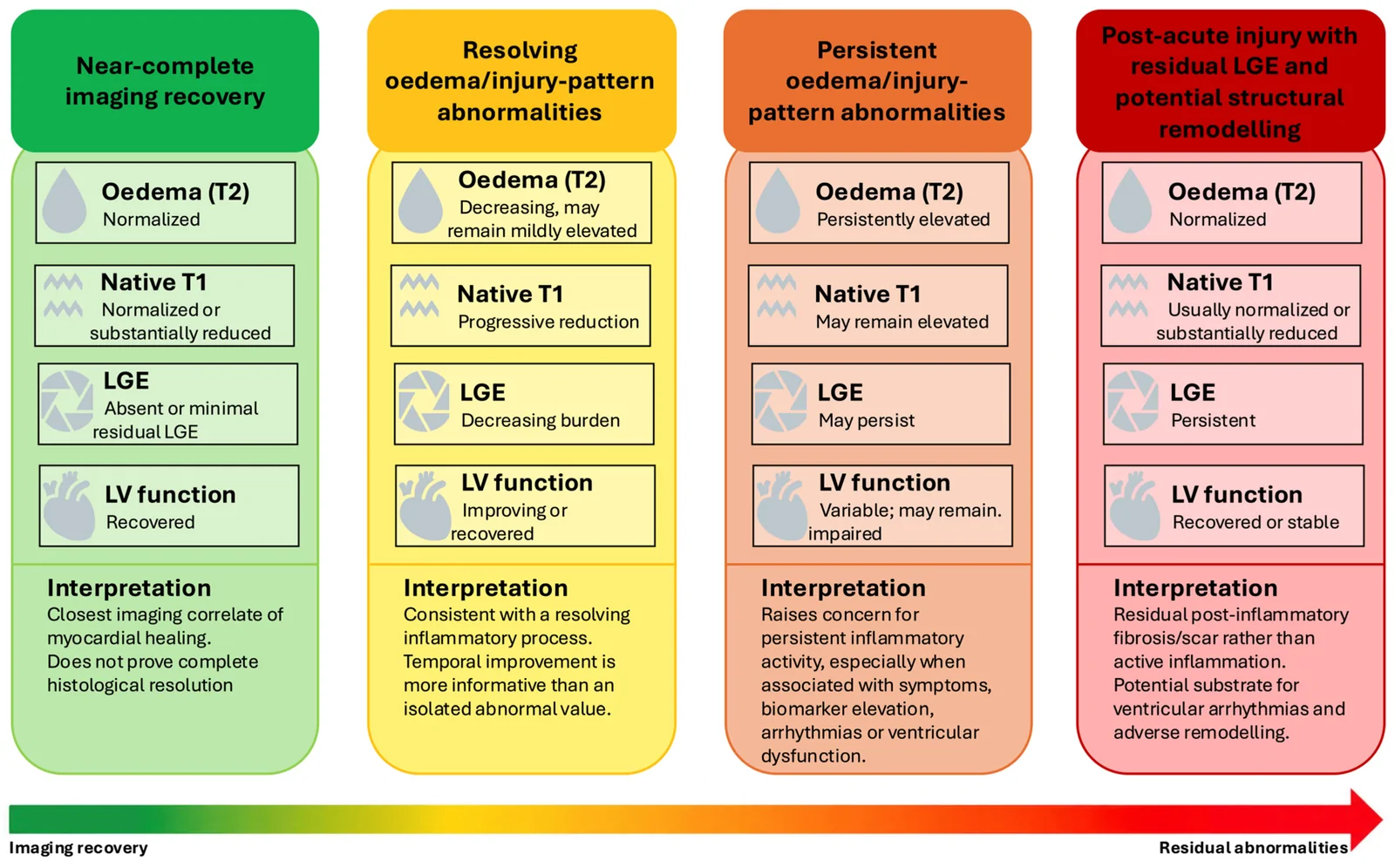
The proposed longitudinal CMR patterns, as a conceptual framework, and their key imaging characteristics. Note: These patterns represent a conceptual framework based on the longitudinal evolution of CMR abnormalities and should not be considered formally validated imaging phenotypes. Individual findings should be interpreted in the context of clinical presentation, biomarkers, ventricular function, and rhythm assessment. The supporting longitudinal evidence derives predominantly from adult patients with conventional acute, clinically uncomplicated myocarditis; extrapolation to specific aetiological or high-risk myocarditis subtypes should therefore be made cautiously. Abbreviations: LGE, late gadolinium enhancement; LV function, left ventricular function.

The proposed longitudinal CMR patterns, as a conceptual framework, and their key imaging characteristics are summarized in Figure 2.

Before describing these longitudinal CMR patterns, their relationship with contemporary disease-stage frameworks should be clarified. The four imaging patterns proposed here are not intended as an alternative classification of myocarditis stages, but rather as a complementary framework describing the myocardial tissue phenotype observed on serial CMR.

The 2025 ESC Guidelines primarily classify myocarditis according to clinical disease duration and course, distinguishing acute, subacute/ongoing, and chronic myocarditis, as well as remission with or without residual abnormalities [2]. More recently, the 2026 EHRA/HFA consensus introduced the “hot”, “hot-to-cold”, and “cold” framework, which emphasizes the evolution from active inflammation toward a progressively fibrotic and potentially arrhythmogenic substrate [65].

These classifications address different dimensions of disease evolution and should therefore not be considered interchangeable. The added value of the present CMR framework is therefore not the introduction of an additional disease-stage classification, but the provision of an imaging-based description of how myocardial tissue abnormalities evolve within and across these established clinical and mechanistic stages. By integrating the longitudinal behaviour of oedema-sensitive markers, native T1/ECV, LGE, and ventricular function, it may help distinguish ongoing or resolving inflammatory injury from residual structural damage—distinctions that cannot be inferred from disease duration or remission status alone. In this sense, the proposed CMR patterns are intended to complement, rather than compete with, the ESC and EHRA/HFA frameworks. Whereas the ESC framework primarily describes clinical time and disease course, and the EHRA/HFA framework emphasizes the evolution of inflammatory and arrhythmic substrate, the present framework focuses specifically on the longitudinal evolution of multiparametric CMR findings. Accordingly, patients within the same ESC temporal stage may exhibit different CMR patterns, and the proposed imaging phenotypes may overlap with, but do not directly correspond to, the EHRA/HFA phases. Importantly, neither framework should be interpreted as implying a strictly linear biological progression, since inflammatory activity, evolving fibrosis, and residual scar may coexist, particularly during the hot-to-cold transition.

The longitudinal evidence supporting these patterns derives predominantly from adults with conventional acute, clinically uncomplicated myocarditis, often with an infarct-like presentation. Accordingly, the proposed framework should not be interpreted as a universal natural-history model applicable to all aetiological and clinical forms of myocarditis. Its applicability to fulminant, giant-cell, eosinophilic, immune-checkpoint-inhibitor-associated, arrhythmic or recurrent myocarditis, cardiac sarcoidosis, and genetically mediated cardiomyopathies presenting with inflammatory “hot phases” remains less well established.

The approximate relationships between these complementary frameworks are summarized in Table 4.

#### 6.2.1. Near-Complete Imaging Recovery

In some patients, follow-up CMR shows near-complete resolution of the abnormalities observed during the acute phase. Oedema-sensitive markers return to normal, T1 abnormalities resolve or substantially decrease, ventricular function recovers, and LGE is absent or limited to small residual areas. This pattern represents the closest CMR correlate of apparent myocardial healing [20,66]. Nevertheless, normalization of CMR findings cannot establish complete histological resolution, as CMR provides an indirect assessment of myocardial inflammation and tissue injury rather than direct histological characterization.

#### 6.2.2. Resolving Edema/Injury-Pattern CMR Abnormalities

A different pattern is observed when CMR abnormalities improve over time without completely normalizing. Oedema-sensitive markers may remain mildly elevated, while T1 abnormalities and LGE progressively decrease. This may be compatible with resolving inflammatory injury, depending on timing and clinical context [37]. In this setting, the evolution of imaging findings may be more informative than whether an individual measurement remains outside the local reference range. A progressive reduction in T2 and T1 values, myocardial mass, and LGE burden may therefore support ongoing myocardial healing despite incomplete imaging normalization [37].

#### 6.2.3. Persistent Oedema/Injury-Pattern CMR Abnormalities

Persistent abnormalities on follow-up CMR, particularly persistently or recurrently elevated T2 in combination with other markers of myocardial injury, may be compatible with persistent inflammatory activity when supported by the appropriate clinical context. However, such findings should not be considered equivalent to biologically or histologically active inflammation. T2 primarily reflects myocardial water content, whereas native T1 and ECV have overlapping determinants and may remain abnormal because of residual extracellular expansion or structural remodelling. CMR therefore identifies a persistent tissue-abnormality pattern but cannot directly demonstrate inflammatory-cell infiltration or establish its underlying cause. Persistent CMR abnormalities alone are insufficient to diagnose chronic active myocarditis or to guide decisions regarding immunosuppressive therapy. When clinically relevant, they should be interpreted together with symptoms, biomarkers, ventricular function, rhythm assessment, and other imaging findings, and may warrant further investigation, including endomyocardial biopsy when histological or aetiological clarification is expected to influence management [2,67,68].

#### 6.2.4. Post-Acute Phase Injury with Residual LGE and Potential Structural Remodelling

Another clinically relevant imaging pattern is characterized by normalization of oedema-sensitive CMR markers and recovery of ventricular function despite persistent LGE. In this setting, residual LGE indicates persistent myocardial tissue abnormalities that may be consistent with fibrosis or structural remodelling, although CMR cannot independently establish their underlying histological substrate. Importantly, normalization of T2 does not necessarily indicate complete histological resolution of myocardial inflammation. Therefore, this pattern should be interpreted as a post-acute imaging phenotype rather than definitive evidence of either established replacement fibrosis or complete inflammatory resolution. Its clinical relevance lies in the potential association of persistent LGE with ventricular arrhythmias and adverse ventricular remodelling, warranting integrated assessment of clinical findings, ventricular function, and arrhythmic risk [59,60,61,62].

#### 6.2.5. Generalizability Across Myocarditis Phenotypes

The longitudinal CMR patterns described above should be interpreted in the context of the populations from which most serial imaging evidence has been derived. Available longitudinal cohorts predominantly include adults with clinically stable, uncomplicated acute myocarditis, often with an infarct-like presentation, whereas evidence describing serial CMR evolution in specific aetiological or high-risk phenotypes remains considerably more limited [2]. Consequently, the relative frequency, timing, and prognostic significance of the proposed patterns may not be uniform across the broader spectrum of myocarditis.

Fulminant myocarditis and specific inflammatory forms such as giant-cell, eosinophilic, immune-checkpoint-inhibitor-associated myocarditis, and cardiac sarcoidosis may exhibit distinct clinical, imaging, and biological profiles [2]. Importantly, CMR itself may provide aetiological clues: for example, eosinophilic myocarditis may show multifocal LGE with frequent subendocardial involvement, whereas CMR abnormalities in immune-checkpoint-inhibitor-associated myocarditis may be less consistently expressed and strongly influenced by imaging timing [69,70,71,72]. Therefore, CMR findings in these settings should not be interpreted solely as markers of temporal disease stage.

Similarly, recurrent or arrhythmic myocarditis and genetically mediated cardiomyopathies presenting with inflammatory “hot phases” may follow a non-linear course characterized by recurrent myocardial injury and progressive or persistent scar rather than a simple unidirectional transition from inflammation to healing [73]. Desmosomal gene variants, particularly DSP variants, have been associated with recurrent myocarditis, ventricular arrhythmias, and a greater extent of LGE at follow-up, further supporting the need to consider an underlying genetic substrate in selected myocarditis-like presentations [74].

Accordingly, the four proposed patterns should be regarded primarily as an imaging-based vocabulary for describing longitudinal tissue changes, with the strongest supporting evidence currently applying to conventional acute myocarditis. Their applicability and prognostic meaning in specific aetiological subgroups require dedicated longitudinal validation.

## 7. Beyond Diagnosis: The Evolving Role of CMR

Longitudinal imaging may identify disease trajectories that are not typical of self-limited myocarditis. Persistent or recurrent inflammatory abnormalities, progressive ventricular dysfunction, or an evolving arrhythmic or structural phenotype should raise suspicion of an underlying disease process and may prompt further diagnostic evaluation, including genetic testing or EMB when clinically appropriate [3,4]. This is particularly relevant for genetically mediated cardiomyopathies that may present with myocarditis-like inflammatory episodes, highlighting the increasing integration of imaging, genetics, and pathology in the evaluation of patients with recurrent or persistent myocardial inflammation [73,75].

In patients already receiving immunomodulatory therapy, serial imaging may provide complementary information on treatment response. Changes in CMR markers of myocardial inflammation, particularly T2, together with recovery of ventricular function and, when available, reduction in metabolic activity on FDG-PET, may help assess the evolution of disease activity during treatment. However, no imaging parameter has yet been validated as a specific surrogate of therapeutic response in myocarditis, and imaging findings should therefore be interpreted together with the clinical course, biomarkers, and, when indicated, histological findings [38].

## 8. Future Directions and Knowledge Gaps

An important future application of CMR may be its potential contribution to therapeutic phenotyping, including the identification of imaging patterns that could eventually help select patients for specific treatments or monitor treatment response. At present, however, longitudinal CMR has not been validated as a treatment-selection biomarker in myocarditis, and no established therapeutic algorithm should be based on serial CMR findings alone. These applications therefore remain investigational and require prospective validation in imaging-guided therapeutic trials [2].

Standardization of parametric mapping, faster and potentially contrast-free protocols, and improved characterization of the transition from active inflammation to healing and residual scar may enhance longitudinal assessment [14,38].

Integration of CMR with other imaging modalities, biomarkers, genetics, and pathology could further refine disease phenotyping, particularly in patients with persistent or recurrent myocardial injury. In parallel, radiomics and artificial intelligence may allow extraction of quantitative imaging features beyond conventional visual assessment [16,76].

Moreover, one of the most promising applications of CMR may be its contribution to therapeutic phenotyping, with imaging used not only to characterize disease but also to identify patients more likely to benefit from specific treatments and to monitor their response. However, these applications remain largely investigational and will require standardized protocols and prospective validation in imaging-guided therapeutic trials [2].

Despite major advances, important gaps remain in the longitudinal interpretation of CMR findings in myocarditis. The natural evolution of T1 and T2 abnormalities, the significance of persistent T2 elevation, and the transition from acute LGE to established scar are not yet fully defined [2]. Although observational studies support the prognostic value of follow-up imaging, it remains uncertain whether serial CMR provides incremental information sufficient to guide therapeutic, arrhythmic, or return-to-exercise decisions [65]. Further studies are also needed to establish whether multimodality imaging can reliably distinguish persistent inflammation from inactive scar and self-limited myocarditis from inflammatory presentations of genetic cardiomyopathy [73]. Ultimately, prospective studies should determine whether imaging-guided management translates into improved clinical outcomes.

## 9. Conclusions

Cardiovascular imaging has substantially changed the diagnosis and longitudinal assessment of myocarditis. Yet myocarditis is not a static condition. Oedema, myocardial injury, ventricular dysfunction, and subsequent repair or fibrosis may evolve at different rates, and improvement in symptoms, biomarkers, and ventricular function does not necessarily coincide with normalization of myocardial tissue abnormalities.

CMR is particularly well suited to capture these changes. T2-sensitive imaging, native T1, ECV, and LGE provide complementary information, but their significance depends on when imaging is performed. This is especially relevant for LGE, which in the acute phase may reflect different components of myocardial injury and may regress during recovery. In contrast, LGE persisting after resolution of oedema is more consistent with residual CMR-detectable tissue abnormalities, although it should not be regarded as direct evidence of histologically established fibrosis. Follow-up CMR should therefore be interpreted in terms of how findings have changed from the acute phase, rather than simply according to whether abnormalities are still present [36,38,77].

Serial imaging may help recognize different patterns of recovery, from resolution of inflammatory injury to persistent abnormalities or residual scar. Although these patterns still require prospective validation, they may provide a useful framework for integrating CMR with the clinical course, biomarkers, ventricular function, and rhythm assessment. Importantly, these CMR patterns should not be interpreted as discrete disease stages, but as imaging phenotypes that may occur within or across established clinical and mechanistic stages, reflecting the imperfect temporal coupling between clinical recovery, inflammatory activity, and structural myocardial healing. This may be particularly relevant when recovery is incomplete or atypical and further investigation, including molecular imaging, genetic testing, or EMB, is being considered.

The value of imaging in myocarditis may therefore increasingly lie in its ability to follow the disease over time. A better understanding of how myocardial abnormalities evolve could refine follow-up, risk assessment, and return-to-exercise decisions and, in the future, may help identify patients who could benefit from more targeted diagnostic or therapeutic strategies.

**Figure 3:**
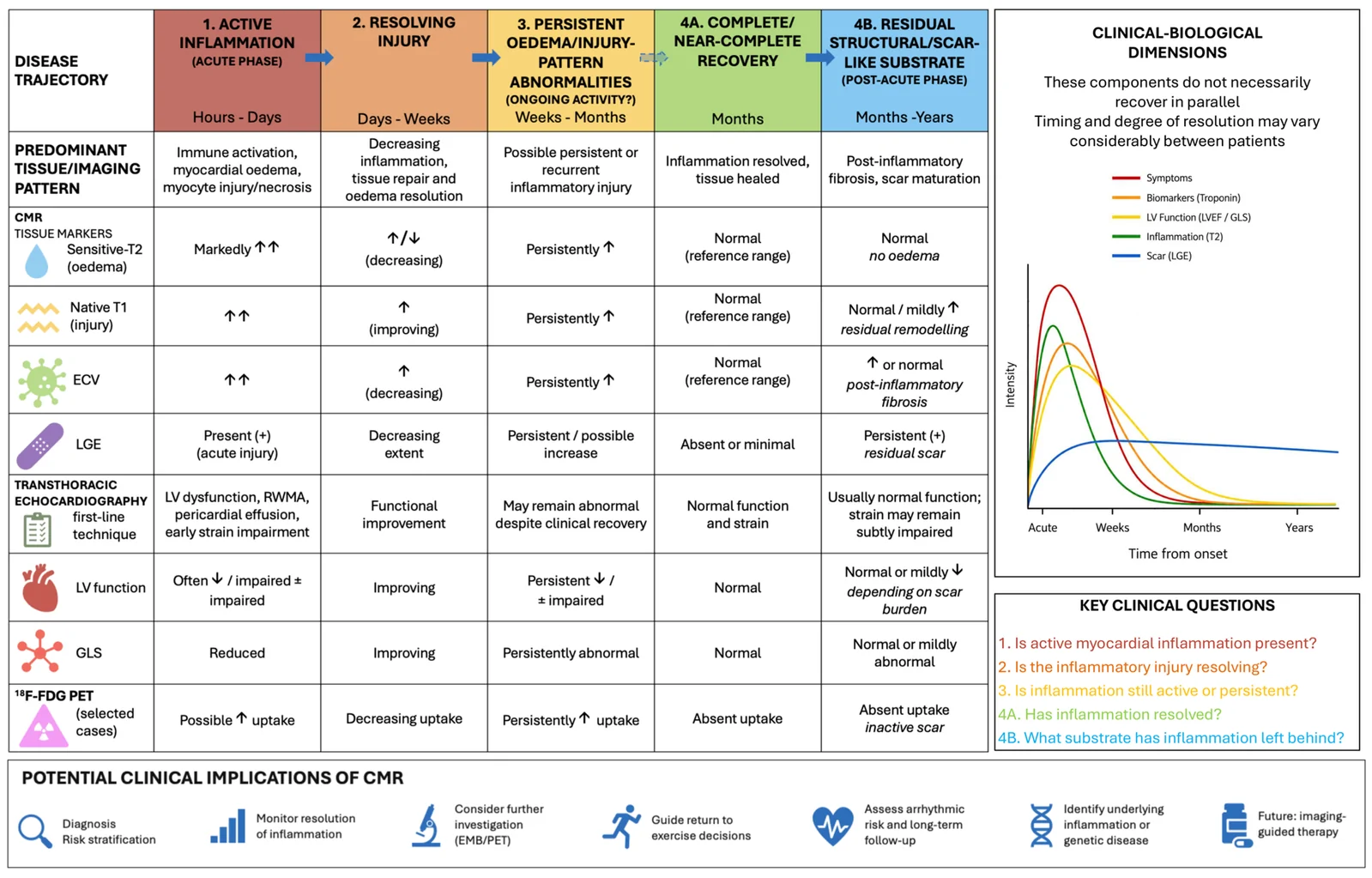
Central Illustration explaining the longitudinal imaging trajectory of myocarditis. The figure illustrates the temporal evolution of CMR, echocardiographic, and complementary findings across the longitudinal course of conventional acute myocarditis. The illustrated tissue patterns represent imaging-based interpretations and should not be considered direct evidence of histologically active inflammation or fibrosis. In particular, persistent CMR abnormalities may be compatible with ongoing inflammatory activity in the appropriate clinical context but cannot establish it in isolation. On the right, the clinical-biological components of recovery are illustrated: they do not necessarily evolve in parallel, and their timing and degree of resolution may vary considerably between patients. The second panel on the right highlights the key clinical questions that may guide imaging interpretation across the disease trajectory. The lower part of the figure summarizes the potential clinical applications of longitudinal CMR, from diagnosis and risk stratification to disease monitoring, return-to-exercise assessment, and future imaging-guided management. The proposed trajectories are conceptual and should not be interpreted as fixed or formally validated phenotypes. Moreover, they derive predominantly from adult patients with conventional acute, clinically uncomplicated myocarditis; extrapolation to specific aetiological, high-risk, recurrent, paediatric, or genetically mediated forms should therefore be made cautiously. Abbreviations: CMR, cardiovascular magnetic resonance; ECV, extracellular volume; LGE, late gadolinium enhancement; LV function, left ventricular function; GLS, global longitudinal strain; ^18^F-FDG-PET, ^18^F-fluorodeoxyglucose positron emission tomography; RWMA, regional wall motion abnormalities; EMB, endomyocardial biopsy; PET, positron emission tomography.

As summarized in the Central Illustration (Figure 3), myocarditis should ultimately be viewed as a dynamic disease trajectory rather than a static imaging diagnosis, in which myocardial inflammation, tissue injury, ventricular function, and residual structural damage evolve along partially independent temporal trajectories. Integrating these complementary dimensions over time may help distinguish active inflammation, resolving injury, persistent inflammatory activity, and healed disease with or without a residual scar substrate, thereby shifting the role of cardiovascular imaging from diagnosis at a single time point towards longitudinal disease characterization.

In this evolving framework, the value of cardiovascular imaging lies not only in identifying myocardial involvement, but increasingly in defining where each patient lies along the disease trajectory and what biological substrate remains over time.

## Figures and Tables

**Table 1:** Complementary roles, strengths, and limitations of cardiovascular imaging modalities in myocarditis.

Imaging Modality	Main Target	Strengths	Limitations
Transthoracic echocardiography (TTE) [2,13]	First-line assessment of biventricular function, regional wall motion, haemodynamic, pericardial effusion, and complications. Speckle-tracking echocardiography (STE) allows the assessment of global and regional myocardial deformation and detection of subtle functional abnormalities.	Widely available, rapid, bedside assessment, no ionizing radiation, well suited to serial functional evaluation. May identify subclinical dysfunction despite preserved LVEF, useful for regional and longitudinal functional assessment.	Limited specificity for myocarditis, may be normal in mild or infarct-like presentations, limited tissue characterization. Dependent on image quality and analysis software, limited disease specificity, diagnostic thresholds are not standardized.
Cardiac magnetic resonance (CMR) [2,4,14,15,16]	Comprehensive assessment of ventricular function and myocardial tissue, including oedema, injury, extracellular expansion, and fibrosis/scar. T1/T2 mapping and ECV allow quantitative assessment of myocardial water content and diffuse tissue/extracellular abnormalities.	Reference non-invasive technique for tissue characterization, multiparametric assessment, no ionizing radiation, central role in diagnosis and follow-up. Detects diffuse abnormalities, quantitative, useful for longitudinal assessment, native T1 and T2 can be acquired without contrast.	Limited availability, longer examination, motion/arrhythmia artefacts, gadolinium may be unsuitable in selected patients, findings are not histologically specific. Reference ranges vary with scanner, sequence, and centre; limited biological specificity; ECV requires contrast and haematocrit; thresholds for persistent disease activity remain uncertain.
Cardiac CT/CCTA [1,17]	Exclusion of coronary artery disease, assessment of cardiac anatomy and function, emerging tissue characterization with late iodine enhancement and CT-derived ECV.	Rapid, high spatial resolution, useful when acute coronary syndrome is part of the differential diagnosis, an option when CMR is not feasible.	Ionizing radiation, iodinated contrast, less established tissue characterization than CMR, limited evidence for routine myocarditis assessment.
^18^F-FDG PET [2,9,16]	Assessment of myocardial metabolic inflammatory activity, particularly in selected or diagnostically challenging cases.	Provides complementary information on inflammatory metabolism; may be useful when CMR is unsuitable (limited by irregular rhythm or device-related artefacts, or diagnostically inconclusive), with a potential role in treatment monitoring.	Limited evidence for routine uncomplicated myocarditis, demanding preparation, physiological myocardial uptake may impair interpretation, radiation exposure, limited standardization.
Hybrid PET/CMR [16]	Combined structural, tissue, functional, and metabolic characterization.	Potential simultaneous integration of CMR-defined injury/scar with metabolic inflammatory activity.	Limited availability, high cost and technical complexity; evidence in myocarditis remains limited, and the clinical role is not established.

Abbreviations: CCTA, coronary computed tomography angiography; CMR, cardiovascular magnetic resonance; ECV, extracellular volume; FDG, fluorodeoxyglucose; LVEF, left ventricular ejection fraction; PET, positron emission tomography; STE, speckle-tracking echocardiography; TTE, transthoracic echocardiography.

**Table 2:** Technical considerations for reliable longitudinal CMR assessment.

Domain	Recommended Approach for Serial CMR	Potential Source of Apparent Longitudinal Change
Scanner and field strength	Whenever possible, use the same scanner/platform and magnetic field strength at baseline and follow-up.	Scanner/platform or field-strength changes may alter absolute T1/T2 values.
Sequences and reconstruction	Maintain the same pulse sequences, acquisition parameters, reconstruction methods, and preferably software versions. Document hardware/software upgrades.	Sequence or reconstruction changes may mimic biological variation.
Acquisition conditions	Reproduce cardiac phase, slice position/orientation, breath-holding strategy, and motion-correction approach as closely as possible.	Differences in cardiac phase, positioning, motion, or partial-volume effects may affect quantitative measurements.
ROI and post-processing	Use consistent myocardial segmentation, ROI strategy, contouring conventions, and post-processing software/methodology.	Changes in ROI placement, segmentation, or software may influence T1, T2, ECV, and LGE quantification.
ECV	Use comparable native/post-contrast T1 methodology, contrast-agent type and dose, and post-contrast timing; obtain contemporaneous haematocrit whenever possible.	Differences in contrast protocol, timing, or haematocrit may alter calculated ECV independently of tissue change.
LGE	Use comparable gadolinium dose, acquisition timing, sequence/reconstruction, and segmentation/quantification method.	Different thresholding, image contrast, or segmentation may produce apparent changes in LGE extent.
Interpretation of change	Interpret serial differences in relation to the precision and scan–rescan reproducibility of the specific technique; favour concordant multiparametric changes.	Small differences within expected measurement variability should not be considered evidence of biological improvement or deterioration.

Abbreviations: CMR, cardiovascular magnetic resonance; ECV, extracellular volume; LGE, late gadolinium enhancement; ROI, region of interest. Note: Direct comparison of quantitative CMR values between serial examinations is most reliable when acquisition and post-processing conditions are comparable. When technical conditions differ, absolute changes should be interpreted cautiously and against appropriately validated reference ranges. Small numerical differences should not be considered biologically meaningful unless they exceed the expected measurement variability of the specific technique and are supported by the broader multiparametric and clinical context.

**Table 3:** Principal longitudinal CMR cohorts informing the temporal evolution of myocarditis.

Study	Population/Phenotype	N	Baseline/Follow-Up CMR	CMR Protocol	Mapping Availability	LGE Assessment	Main Longitudinal Findings/Clinical Outcomes	Major Limitations
Aquaro et al., 2019 [20]	Multicentre ITAMY subgroup with acute myocarditis; clinically stable patients undergoing serial CMR.	187	Within 1 week of symptom onset; repeat CMR at 6 months; median clinical follow-up 7 years (IQR 6–8).	Cine imaging; oedema-sensitive imaging; LGE.	No parametric mapping reported in the longitudinal analysis.	Presence, distribution and change in LGE extent/number of involved segments; particular attention to mid-wall septal LGE.	At 6 months, oedema persisted in 16%; LGE in 86%. Complete recovery from both oedema and LGE occurred in 11%. LGE without oedema and mid-wall septal LGE independently predicted cardiac events; 22 events occurred during long-term follow-up.	Observational registry subgroup; heterogeneous multicentre acquisition; selected clinically stable population; no serial T1/T2 mapping; 6-month interval does not define earlier kinetics of recovery.
Pieroni et al., 2024 [19]	Consecutive patients with uncomplicated acute myocarditis; predominantly young male population with mainly infarct-like presentation.	33	Presentation; 3 months; 12 months; subsequent clinical/Holter/echo follow-up (85 ± 15 months).	Cine imaging; oedema assessment; LGE; LV mass and function.	No parametric mapping reported.	LGE quantified as absolute mass (g) and percentage of LV mass; serial LGE burden assessed after oedema resolution.	By 3 months, oedema, LGE burden and LV mass decreased, and LVEF improved. Oedema persisted in 21% at 3 months but resolved by 12 months; LGE showed no further significant change after 3 months. Persistent ventricular arrhythmias occurred in 15%; LGE extent at 3 and 12 months was associated with arrhythmic persistence.	Small cohort; uncomplicated phenotype with limited generalizability to severe or non-infarct-like myocarditis; selected population; no parametric mapping; limited number of arrhythmic events.
Bohnen et al., 2017 [38]	Strictly defined acute myocarditis; comparison with 27 healthy controls.	48	Acute stage; 3 months (*n* = 39); 12 months (*n* = 21).	1.5-T CMR; conventional Lake Louise sequences plus native/post-contrast T1 and T2 mapping.	Yes: MOLLI T1 mapping, GraSE T2 mapping and ECV.	Conventional LGE included within the Lake Louise protocol; serial tissue characterization focused primarily on T1, T2 and ECV.	Native T1, T2 and ECV declined during healing. Global and regional native T1/T2 differentiated acute from healed myocarditis, supporting quantitative mapping as a marker of temporal resolution of inflammatory injury.	Single-centre study; modest sample size; substantial attrition at 12 months (21/48); diagnostic rather than outcome-powered study; mapping values are sequence- and site-dependent.
Luetkens et al., 2016 [14]	Prospective cohort with clinically suspected acute myocarditis; 45 healthy controls.	24	Baseline 2.6 ± 1.9 days after admission; follow-up at 2.4 ± 0.6 weeks (*n* = 11), 5.5 ± 1.3 weeks (*n* = 20), and 16.2 ± 9.9 weeks (*n* = 24).	Conventional Lake Louise criteria; cine imaging; T1 mapping; T2 mapping; ECV; LGE.	Yes: native/post-contrast T1, T2 mapping and ECV.	Segmented inversion-recovery gradient-echo LGE; enhanced myocardium quantified using a ≥3-SD threshold above reference normal myocardium.	Inflammatory CMR parameters decreased consistently over time. Native T1, T2, ECV, oedema and LGE abnormalities were most pronounced acutely and progressively normalized, demonstrating the strong dependence of CMR sensitivity on imaging timing.	Small single-centre cohort; clinically suspected rather than biopsy-proven myocarditis; unequal numbers across early follow-up time points; short imaging follow-up; no long-term prognostic endpoint.

Note: The table summarizes the principal serial-CMR cohorts directly informing the longitudinal framework discussed in this review. The studies differ substantially in sample size, clinical phenotype, imaging intervals, acquisition protocols and availability of parametric mapping; their findings should therefore not be interpreted as providing equivalent evidentiary weight. Abbreviations: CMR, cardiovascular magnetic resonance; ECV, extracellular volume; GraSE, gradient- and spin-echo; IQR, interquartile range; ITAMY, ITAlian study in MYocarditis; LGE, late gadolinium enhancement; LV, left ventricular; LVEF, left ventricular ejection fraction; MOLLI, modified Look-Locker inversion recovery.

**Table 4:** Conceptual relationship between the proposed longitudinal CMR patterns and contemporary myocarditis disease-stage frameworks. The proposed correspondences are conceptual and should not be interpreted as diagnostic equivalences. ESC terminology primarily reflects disease duration and clinical course, the EHRA/HFA framework emphasizes the evolution of inflammatory and arrhythmic substrate, whereas the proposed CMR patterns describe the longitudinal evolution of myocardial tissue abnormalities on serial imaging. Considerable overlap between categories may therefore occur. Importantly, the EHRA/HFA phases represent predominant and potentially overlapping biological and electrophysiological states rather than strictly sequential chronological categories. Arrhythmic risk cannot be inferred from the corresponding CMR pattern alone and requires integration of the presenting and subsequent arrhythmic phenotype, ventricular function, LGE burden and topology, clinical risk markers, and, when appropriate, genetic background.

Proposed Longitudinal CMR Pattern	Main CMR Features	Relationship with 2025 ESC Disease Stage/Remission Terminology	Relationships with 2026 EHRA/HFA Framework
Near-complete imaging recovery	Resolution or near-resolution of oedema-sensitive abnormalities and other markers of myocardial injury; absent or minimal residual LGE.	Most consistent with remission without residuals, when accompanied by clinical, biomarker, and ECG normalization.	Resolution of the “hot” phase without an established residual scar substrate.
Resolving inflammatory injury	Progressive reduction in T2/native T1 abnormalities and/or LGE burden, indicating incomplete but ongoing tissue recovery.	May occur during acute or subacute/ongoing myocarditis and during transition toward remission.	May overlap with the “hot-to-cold” transition, although resolving CMR abnormalities do not necessarily imply an arrhythmogenic substrate.
Persistent oedema/injury-pattern CMR abnormalities	Persistent or recurrent oedema-sensitive abnormalities, particularly elevated T2, with associated markers of myocardial injury.	Most relevant to subacute/ongoing or chronic myocarditis, although temporal criteria alone do not establish persistent inflammation.	May be compatible with a persistent ‘hot’ or ‘hot-to-cold’ phenotype when supported by the clinical, biomarker, rhythm, and/or multimodal imaging context; CMR abnormalities alone do not establish persistent inflammatory activity.
Post-acute phase with residual LGE and potential structural remodelling	Resolution of oedema-sensitive abnormalities with persistent LGE ± residual structural/functional remodelling.	Most consistent with remission with residuals; may also be encountered in chronic/post-inflammatory states.	Most closely corresponds to the “cold” phase, characterized by residual tissue abnormalities potentially consistent with fibrosis or structural remodelling.

Abbreviations: CMR, cardiovascular magnetic resonance; ECG, electrocardiogram; ESC, European Society of Cardiology; EHRA, European Heart Rhythm Association; HFA, Heart Failure Association; LGE, late gadolinium enhancement.

## Data Availability

No new data were created or analyzed in this study. Data sharing is not applicable to this article.

## Abbreviations

The following abbreviations are used in this manuscript:

AMO	Acute myocardial oedema
CMR	Cardiovascular magnetic resonance
CT	Computed tomography
ECG	Electrocardiogram
ECV	Extracellular volume
EMB	Endomyocardial biopsy
ESC	European Society of Cardiology
FDG	Fluorodeoxyglucose
FDG-PET	^18^F-fluorodeoxyglucose positron emission tomography
ICD	Implantable cardioverter-defibrillator
LGE	Late gadolinium enhancement
LLC	Lake Louise Criteria
LV	Left ventricular/left ventricle
LV-GLS	Left ventricular global longitudinal strain
LVEF	Left ventricular ejection fraction
PET	Positron emission tomography
PET-CMR	Positron emission tomography–cardiovascular magnetic resonance
STIR	Short-tau inversion recovery
TTE	Transthoracic echocardiography

## References

[1] Ammirati E., Frigerio M., Adler E.D., Basso C., Birnie D.H., Brambatti M., Friedrich M.G., Klingel K., Lehtonen J., Moslehi J.J. (2020). Management of Acute Myocarditis and Chronic Inflammatory Cardiomyopathy: An Expert Consensus Document. Circ. Heart Fail..

[2] Schulz-Menger J., Collini V., Gröschel J., Adler Y., Brucato A., Christian V., Ferreira V.M., Gandjbakhch E., Heidecker B., Kerneis M. (2025). 2025 ESC Guidelines for the management of myocarditis and pericarditis. Eur. Heart J..

[3] Drazner M.H., Bozkurt B., Cooper L.T., Aggarwal N.R., Basso C., Bhave N.M., Caforio A.L.P., Ferreira V.M., Heidecker B., Writing Committee (2025). 2024 ACC Expert Consensus Decision Pathway on Strategies and Criteria for the Diagnosis and Management of Myocarditis: A Report of the American College of Cardiology Solution Set Oversight Committee. J. Am. Coll. Cardiol..

[4] Ferreira V.M., Schulz-Menger J., Holmvang G., Kramer C.M., Carbone I., Sechtem U., Kindermann I., Gutberlet M., Cooper L.T., Liu P. (2018). Cardiovascular Magnetic Resonance in Nonischemic Myocardial Inflammation: Expert Recommendations. J. Am. Coll. Cardiol..

[5] Muscogiuri G., Guaricci A.I., Cau R., Saba L., Senatieri A., Chierchia G., Pontone G., Volpato V., Palmisano A., Esposito A. (2022). Multimodality imaging in acute myocarditis. J. Clin. Ultrasound.

[6] Adeboye A., Alkhatib D., Butt A., Yedlapati N., Garg N. (2022). A Review of the Role of Imaging Modalities in the Evaluation of Viral Myocarditis with a Special Focus on COVID-19-Related Myocarditis. Diagnostics.

[7] Budoff M.J., Dowe D., Jollis J.G., Gitter M., Sutherland J., Halamert E., Scherer M., Bellinger R., Martin A., Benton R. (2008). Diagnostic performance of 64-multidetector row coronary computed tomographic angiography for evaluation of coronary artery stenosis in individuals without known coronary artery disease: Results from the prospective multicenter ACCURACY (Assessment by Coronary Computed Tomographic Angiography of Individuals Undergoing Invasive Coronary Angiography) trial. J. Am. Coll. Cardiol..

[8] Steffani S., Piscione M., Gaudio D., Meghnagi G., Montella V., Fiorini F., Micillo A., Tagliati C., Asmundo L., Manenti G. (2026). Diagnostic Value and Operational Recommendations for Late Iodine Enhancement and ECV Quantification in Single-Energy Computed Tomography: A Narrative Review. Diagnostics.

[9] Peretto G., Busnardo E., Ferro P., Palmisano A., Vignale D., Esposito A., De Luca G., Campochiaro C., Sartorelli S., De Gaspari M. (2022). Clinical Applications of FDG-PET Scan in Arrhythmic Myocarditis. JACC Cardiovasc. Imaging.

[10] Caforio A.L., Pankuweit S., Arbustini E., Basso C., Gimeno-Blanes J., Felix S.B., Fu M., Heliö T., Heymans S., Jahns R. (2013). Current state of knowledge on aetiology, diagnosis, management, and therapy of myocarditis: A position statement of the European Society of Cardiology Working Group on Myocardial and Pericardial Diseases. Eur. Heart J..

[11] Nensa F., Kloth J., Tezgah E., Poeppel T.D., Heusch P., Goebel J., Nassenstein K., Schlosser T. (2018). Feasibility of FDG-PET in myocarditis: Comparison to CMR using integrated PET/MRI. J. Nucl. Cardiol..

[12] Peretto G., Sala S., Basso C., Rizzo S., Radinovic A., Frontera A., Limite L.R., Paglino G., Bisceglia C., De Luca G. (2020). Inflammation as a Predictor of Recurrent Ventricular Tachycardia After Ablation in Patients with Myocarditis. J. Am. Coll. Cardiol..

[13] Kandels J., Richter S., Hagendorff A., Kragholm K., Tayal B., Laufs U., Denecke T., Stöbe S. (2024). Comparison of left ventricular deformation abnormalities by echocardiography with cardiac magnetic resonance imaging in patients with acute myocarditis and preserved left ventricular ejection fraction. Front Cardiovasc. Med..

[14] Luetkens J.A., Homsi R., Dabir D., Kuetting D.L., Marx C., Doerner J., Schlesinger-Irsch U., Andrié R., Sprinkart A.M., Schmeel F.C. (2016). Comprehensive Cardiac Magnetic Resonance for Short-Term Follow-Up in Acute Myocarditis. J. Am. Heart Assoc..

[15] Lurz J.A., Luecke C., Lang D., Besler C., Rommel K.P., Klingel K., Kandolf R., Adams V., Schöne K., Hindricks G. (2018). CMR-Derived Extracellular Volume Fraction as a Marker for Myocardial Fibrosis: The Importance of Coexisting Myocardial Inflammation. JACC Cardiovasc. Imaging.

[16] Caobelli F., Cabrero J.B., Galea N., Haaf P., Loewe C., Luetkens J.A., Muscogiuri G., Francone M. (2023). Cardiovascular magnetic resonance (CMR) and positron emission tomography (PET) imaging in the diagnosis and follow-up of patients with acute myocarditis and chronic inflammatory cardiomyopathy: A review paper with practical recommendations on behalf of the European Society of Cardiovascular Radiology (ESCR). Int. J. Cardiovasc. Imaging.

[17] Bartoli A., Gnasso C., Palmisano A., Bettinelli A., Vignale D., Esposito A. (2025). Myocardial Characterization on CT: Late Iodine Enhancement and Extracellular Volume. Echocardiography.

[18] Berg J., Kottwitz J., Baltensperger N., Kissel C.K., Lovrinovic M., Mehra T., Scherff F., Schmied C., Templin C., Lüscher T.F. (2017). Cardiac Magnetic Resonance Imaging in Myocarditis Reveals Persistent Disease Activity Despite Normalization of Cardiac Enzymes and Inflammatory Parameters at 3-Month Follow-Up. Circ. Heart Fail..

[19] Pieroni M., Ciabatti M., Zocchi C., Tavanti V., Camporeale A., Saletti E., Fumagalli C., Venezia D., Lombardi M., Olivotto I. (2024). Optimal timing of follow-up cardiac magnetic resonance in patients with uncomplicated acute myocarditis. Int. J. Cardiol..

[20] Aquaro G.D., Ghebru Habtemicael Y., Camastra G., Monti L., Dellegrottaglie S., Moro C., Lanzillo C., Scatteia A., Di Roma M., Pontone G. (2019). “Cardiac Magnetic Resonance” Working Group of the Italian Society of Cardiology. Prognostic Value of Repeating Cardiac Magnetic Resonance in Patients with Acute Myocarditis. J. Am. Coll. Cardiol..

[21] Law Y.M., Lal A.K., Chen S., Čiháková D., Cooper L.T., Deshpande S., Godown J., Grosse-Wortmann L., Robinson J.D., Towbin J.A. (2021). Diagnosis and Management of Myocarditis in Children: A Scientific Statement from the American Heart Association. Circulation.

[22] Fratz S., Chung T., Greil G.F., Samyn M.M., Taylor A.M., Valsangiacomo Buechel E.R., Yoo S.J., Powell A.J. (2013). Guidelines and protocols for cardiovascular magnetic resonance in children and adults with congenital heart disease: SCMR expert consensus group on congenital heart disease. J. Cardiovasc. Magn. Reson..

[23] Messroghli D.R., Moon J.C., Ferreira V.M., Grosse-Wortmann L., He T., Kellman P., Mascherbauer J., Nezafat R., Salerno M., Schelbert E.B. (2017). Clinical recommendations for cardiovascular magnetic resonance mapping of T1, T2, T2* and extracellular volume: A consensus statement by the Society for Cardiovascular Magnetic Resonance (SCMR) endorsed by the European Association for Cardiovascular Imaging (EACVI). J. Cardiovasc. Magn. Reson..

[24] Lurz P., Eitel I., Adam J., Steiner J., Grothoff M., Desch S., Fuernau G., de Waha S., Sareban M., Luecke C. (2012). Diagnostic performance of CMR imaging compared with EMB in patients with suspected myocarditis. JACC Cardiovasc. Imaging.

[25] Hiramitsu S., Morimoto S., Kato S., Uemura A., Kubo N., Kimura K., Sugiura A., Itoh T., Hishida H. (2001). Transient ventricular wall thickening in acute myocarditis: A serial echocardiographic and histopathologic study. Jpn. Circ. J..

[26] Zagrosek A., Wassmuth R., Abdel-Aty H., Rudolph A., Dietz R., Schulz-Menger J. (2008). Relation between myocardial edema and myocardial mass during the acute and convalescent phase of myocarditis--a CMR study. J. Cardiovasc. Magn. Reson..

[27] Weber L., Sokolska J.M., Nadarevic T., Karolyi M., Baessler B., Fischer X., Sokolski M., von Spiczak J., Polacin M., Matziris I. (2022). Impact of myocardial injury on regional left ventricular function in the course of acute myocarditis with preserved ejection fraction: Insights from segmental feature tracking strain analysis using cine cardiac MRI. Int. J. Cardiovasc. Imaging.

[28] Scatteia A., Baritussio A., Bucciarelli-Ducci C. (2017). Strain imaging using cardiac magnetic resonance. Heart Fail. Rev..

[29] Isaak A., Kravchenko D., Mesropyan N., Endler C., Bischoff L.M., Vollbrecht T., Thomas D., Dabir D., Zimmer S., Attenberger U. (2022). Layer-specific Strain Analysis with Cardiac MRI Feature Tracking in Acute Myocarditis. Radiol. Cardiothorac. Imaging.

[30] Lagan J., Schmitt M., Miller C.A. (2018). Clinical applications of multi-parametric CMR in myocarditis and systemic inflammatory diseases. Int. J. Cardiovasc. Imaging.

[31] Friedrich M.G., Sechtem U., Schulz-Menger J., Holmvang G., Alakija P., Cooper L.T., White J.A., Abdel-Aty H., Gutberlet M., Prasad S. (2009). International Consensus Group on Cardiovascular Magnetic Resonance in Myocarditis. Cardiovascular magnetic resonance in myocarditis: A JACC White Paper. J. Am. Coll. Cardiol..

[32] Sinigiani G., De Michieli L., De Conti G., Ricci F., De Lazzari M., Migliore F., Perazzolo Marra M., Zorzi A., Corrado D., Cipriani A. (2023). Cardiac Magnetic Resonance-Detected Acute Myocardial Edema as Predictor of Favourable Prognosis: A Comprehensive Review. J. Cardiovasc. Dev. Dis..

[33] Eitel I., Friedrich M.G. (2011). T2-weighted cardiovascular magnetic resonance in acute cardiac disease. J. Cardiovasc. Magn. Reson..

[34] Scallan J., Huxley V.H., Korthuis R.J. (2010). Capillary Fluid Exchange: Regulation, Functions, and Pathology.

[35] Rajiah P., Kirsch J., Bolen M.A., Batlle J.C., Brown R.K.J., Francois C.J., Galizia M.S., Hanneman K., Inacio J.R., Expert Panel on Cardiac Imaging (2021). ACR Appropriateness Criteria^®^ Nonischemic Myocardial Disease with Clinical Manifestations (Ischemic Cardiomyopathy Already Excluded). J. Am. Coll. Radiol..

[36] Moon J.C., Messroghli D.R., Kellman P., Piechnik S.K., Robson M.D., Ugander M., Gatehouse P.D., Arai A.E., Friedrich M.G., Neubauer S. (2013). Myocardial T1 mapping and extracellular volume quantification: A Society for Cardiovascular Magnetic Resonance (SCMR) and CMR Working Group of the European Society of Cardiology consensus statement. J. Cardiovasc. Magn. Reson..

[37] Eichhorn C., Greulich S., Bucciarelli-Ducci C., Sznitman R., Kwong R.Y., Gräni C. (2022). Multiparametric Cardiovascular Magnetic Resonance Approach in Diagnosing, Monitoring, and Prognostication of Myocarditis. JACC Cardiovasc. Imaging.

[38] Bohnen S., Radunski U.K., Lund G.K., Ojeda F., Looft Y., Senel M., Radziwolek L., Avanesov M., Tahir E., Stehning C. (2017). Tissue characterization by T1 and T2 mapping cardiovascular magnetic resonance imaging to monitor myocardial inflammation in healing myocarditis. Eur. Heart J. Cardiovasc. Imaging.

[39] De Lazzari M., Zorzi A., Baritussio A., Siciliano M., Migliore F., Susana A., Giorgi B., Lacognata C., Iliceto S., Perazzolo Marra M. (2016). Relationship between T-wave inversion and transmural myocardial edema as evidenced by cardiac magnetic resonance in patients with clinically suspected acute myocarditis: Clinical and prognostic implications. J. Electrocardiol..

[40] Bohbot Y., Garot J., Hovasse T., Unterseeh T., Di Lena C., Boukefoussa W., Tawa C., Renard C., Limouzineau I., Duhamel S. (2022). Clinical and Cardiovascular Magnetic Resonance Predictors of Early and Long-Term Clinical Outcome in Acute Myocarditis. Front Cardiovasc. Med..

[41] McLellan A.J., McKenzie S.C., Taylor A.J. (2012). Cardiac magnetic resonance imaging predicts recovery of left ventricular function in acute onset cardiomyopathy. Heart Lung Circ..

[42] Aquaro G.D., Perfetti M., Camastra G., Monti L., Dellegrottaglie S., Moro C., Pepe A., Todiere G., Lanzillo C., Scatteia A. (2017). Cardiac Magnetic Resonance Working Group of the Italian Society of Cardiology. Cardiac MR with Late Gadolinium Enhancement in Acute Myocarditis with Preserved Systolic Function: ITAMY Study. J. Am. Coll. Cardiol..

[43] Georgiopoulos G., Figliozzi S., Sanguineti F., Aquaro G.D., di Bella G., Stamatelopoulos K., Chiribiri A., Garot J., Masci P.G., Ismail T.F. (2021). Prognostic Impact of Late Gadolinium Enhancement by Cardiovascular Magnetic Resonance in Myocarditis: A Systematic Review and Meta-Analysis. Circ. Cardiovasc. Imaging.

[44] Mahrholdt H., Goedecke C., Wagner A., Meinhardt G., Athanasiadis A., Vogelsberg H., Fritz P., Klingel K., Kandolf R., Sechtem U. (2004). Cardiovascular magnetic resonance assessment of human myocarditis: A comparison to histology and molecular pathology. Circulation.

[45] Grün S., Schumm J., Greulich S., Wagner A., Schneider S., Bruder O., Kispert E.M., Hill S., Ong P., Klingel K. (2012). Long-term follow-up of biopsy-proven viral myocarditis: Predictors of mortality and incomplete recovery. J. Am. Coll. Cardiol..

[46] Imazio M., Angelico G., Andriani M., Lobetti-Bodoni L., Davini O., Giustetto C., Rinaldi M. (2018). Prevalence and Prognostic Impact of Septal Late Gadolinium Enhancement in Acute Myocarditis with or Without Preserved Left Ventricular Function. Am. J. Cardiol..

[47] Sanguineti F., Garot P., Mana M., O’h-Ici D., Hovasse T., Unterseeh T., Louvard Y., Troussier X., Morice M.C., Garot J. (2015). Cardiovascular magnetic resonance predictors of clinical outcome in patients with suspected acute myocarditis. J. Cardiovasc. Magn. Reson..

[48] Yang F., Wang J., Li W., Xu Y., Wan K., Zeng R., Chen Y. (2020). The prognostic value of late gadolinium enhancement in myocarditis and clinically suspected myocarditis: Systematic review and meta-analysis. Eur. Radiol..

[49] Isaak A., Wirtz J., Kravchenko D., Mesropyan N., Bischoff L.M., Bienert S., Weinhold L., Pieper C.C., Attenberger U., Öztürk C. (2024). Cardiac MRI in infarct-like myocarditis: Transmural extension of late gadolinium enhancement is associated with worse outcomes. Insights Imaging.

[50] Lintingre P.F., Nivet H., Clément-Guinaudeau S., Camaioni C., Sridi S., Corneloup O., Gerbaud E., Coste P., Dournes G., Latrabe V. (2020). High-Resolution Late Gadolinium Enhancement Magnetic Resonance for the Diagnosis of Myocardial Infarction with Nonobstructed Coronary Arteries. JACC Cardiovasc. Imaging.

[51] Kramer C.M., Barkhausen J., Bucciarelli-Ducci C., Flamm S.D., Kim R.J., Nagel E. (2020). Standardized cardiovascular magnetic resonance imaging (CMR) protocols: 2020 update. J. Cardiovasc. Magn. Reson..

[52] Schulz-Menger J., Bluemke D.A., Bremerich J., Flamm S.D., Fogel M.A., Friedrich M.G., Kim R.J., von Knobelsdorff-Brenkenhoff F., Kramer C.M., Pennell D.J. (2020). Standardized image interpretation and post-processing in cardiovascular magnetic resonance—2020 update: Society for Cardiovascular Magnetic Resonance (SCMR): Board of Trustees Task Force on Standardized Post-Processing. J. Cardiovasc. Magn. Reson..

[53] Böttcher B., Lorbeer R., Stöcklein S., Beller E., Lang C.I., Weber M.A., Meinel F.G. (2021). Global and Regional Test-Retest Reproducibility of Native T1 and T2 Mapping in Cardiac Magnetic Resonance Imaging. J. Magn. Reson Imaging.

[54] Meier C., Bietenbeck M., Korthals D., Vehof V., Stalling P., Akyol N., Theofanidou M., Yilmaz A. (2024). Precision of Myocardial T1- and T2-Mapping Using CMR: Prospective Scan-Rescan Approach to Assess Mapping Data Reproducibility. JACC Cardiovasc. Imaging.

[55] Tschöpe C., Ammirati E., Bozkurt B., Caforio A.L.P., Cooper L.T., Felix S.B., Hare J.M., Heidecker B., Heymans S., Hübner N. (2021). Myocarditis and inflammatory cardiomyopathy: Current evidence and future directions. Nat. Rev. Cardiol..

[56] Francone M., Chimenti C., Galea N., Scopelliti F., Verardo R., Galea R., Carbone I., Catalano C., Fedele F., Frustaci A. (2014). CMR sensitivity varies with clinical presentation and extent of cell necrosis in biopsy-proven acute myocarditis. JACC Cardiovasc. Imaging.

[57] Radunski U.K., Lund G.K., Stehning C., Schnackenburg B., Bohnen S., Adam G., Blankenberg S., Muellerleile K. (2014). CMR in patients with severe myocarditis: Diagnostic value of quantitative tissue markers including extracellular volume imaging. JACC Cardiovasc. Imaging.

[58] Priori S.G., Blomström-Lundqvist C., Mazzanti A., Blom N., Borggrefe M., Camm J., Elliott P.M., Fitzsimons D., Hatala R., Hindricks G. (2015). 2015 ESC Guidelines for the management of patients with ventricular arrhythmias and the prevention of sudden cardiac death: The Task Force for the Management of Patients with Ventricular Arrhythmias and the Prevention of Sudden Cardiac Death of the European Society of Cardiology (ESC). Endorsed by: Association for European Paediatric and Congenital Cardiology (AEPC). Eur. Heart J..

[59] Peretto G., Sala S., Rizzo S., De Luca G., Campochiaro C., Sartorelli S., Benedetti G., Palmisano A., Esposito A., Tresoldi M. (2019). Arrhythmias in myocarditis: State of the art. Heart Rhythm.

[60] Peretto G., Sala S., Rizzo S., Palmisano A., Esposito A., De Cobelli F., Campochiaro C., De Luca G., Foppoli L., Dagna L. (2020). Ventricular Arrhythmias in Myocarditis: Characterization and Relationships with Myocardial Inflammation. J. Am. Coll. Cardiol..

[61] Narducci M.L., Ballacci F., Giordano F., Collini V., Imazio M. (2024). Sudden cardiac death after acute myocarditis with arrhythmic presentation: Hunting for risk predictors—A systematic review and meta-analysis. Open Heart.

[62] Imazio M., Collini V., Tomat M., Venturelli F., Spollero G., Gröschel J., Lazarou E., Schulz-Menger J., Lazaros G. (2026). Arrhythmic myocarditis and prevention of sudden cardiac death: Current evidence, risk stratification, and management. Hell. J. Cardiol..

[63] Bryde R.E., Cooper L.T., Fairweather D., Di Florio D.N., Martinez M.W. (2023). Exercise After Acute Myocarditis: When and How to Return to Sports. Cardiol. Clin..

[64] Gallinoro C.M., Scatteia A., Catapano D., Pascale C.E., Russo G., Di Meglio F., Dellegrottaglie S. (2025). 2020 ESC Guidelines on Sports Cardiology: Impact of CMR Criteria on Return-to-Play Clearance After Acute Myocarditis. J. Cardiovasc. Dev. Dis..

[65] Peretto G., Basso C., Berruezo A., Bollano E., Caforio A.L.P., Casella M., De Chillou C., Ebert M., Gati S., Imazio M. (2026). Workup and management of rhythm disorders in myocarditis and inflammatory cardiomyopathy: A clinical consensus statement of the European Heart Rhythm Association and the Heart Failure Association of the ESC, the ESC Working Group on Myocardial & Pericardial Diseases, the European Association of Preventive Cardiology of the ESC, the Heart Rhythm Society, the Asian Pacific Heart Rhythm Society, and the Latin American Heart Rhythm Society. Eur. J. Heart Fail..

[66] Baritussio A., Cheng C.Y., Simeti G., Ocagli H., Lorenzoni G., Giordani A.S., Basso C., Rizzo S., De Gaspari M., Motta R. (2024). CMR Predictors of Favorable Outcome in Myocarditis: A Single-Center Experience. J. Clin. Med..

[67] Krumm P., Brendel J.M., Klingel K., Müller K.A.L., Kübler J., Gräni C., Gawaz M., Nikolaou K., Greulich S. (2022). Using Multiparametric Cardiac Magnetic Resonance to Phenotype and Differentiate Biopsy-Proven Chronic from Healed Myocarditis and Dilated Cardiomyopathy. J. Clin. Med..

[68] Alam W., Hamrouni H., Choneva I., Moini C. (2026). Multiparametric CMR in Myocarditis: A Comprehensive Review of Diagnostic Advances, Prognostic Value, and the Challenge of Genetic Mimics. Biomedicines.

[69] Pöyhönen P., Nordenswan H.K., Lehtonen J., Syväranta S., Shenoy C., Kupari M. (2023). Cardiac magnetic resonance in giant cell myocarditis: A matched comparison with cardiac sarcoidosis. Eur. Heart J. Cardiovasc. Imaging.

[70] Cheng R.K., Kittleson M.M., Beavers C.J., Birnie D.H., Blankstein R., Bravo P.E., Gilotra N.A., Judson M.A., Patton K.K., Rose-Bovino L. (2024). Diagnosis and Management of Cardiac Sarcoidosis: A Scientific Statement from the American Heart Association. Circulation.

[71] Pöyhönen P., Rågback J., Mäyränpää M.I., Nordenswan H.K., Lehtonen J., Shenoy C., Kupari M. (2023). Cardiac magnetic resonance in histologically proven eosinophilic myocarditis. J. Cardiovasc. Magn. Reson..

[72] Zhang L., Awadalla M., Mahmood S.S., Nohria A., Hassan M.Z.O., Thuny F., Zlotoff D.A., Murphy S.P., Stone J.R., Golden D.L.A. (2020). Cardiovascular magnetic resonance in immune checkpoint inhibitor-associated myocarditis. Eur. Heart J..

[73] Bariani R., Rigato I., Cipriani A., Bueno Marinas M., Celeghin R., Basso C., Corrado D., Pilichou K., Bauce B. (2022). Myocarditis-like Episodes in Patients with Arrhythmogenic Cardiomyopathy: A Systematic Review on the So-Called Hot-Phase of the Disease. Biomolecules.

[74] Ammirati E., Raimondi F., Piriou N., Sardo Infirri L., Mohiddin S.A., Mazzanti A., Shenoy C., Cavallari U.A., Imazio M., Aquaro G.D. (2022). Acute Myocarditis Associated with Desmosomal Gene Variants. JACC Heart Fail..

[75] Brandão M., Bariani R., Rigato I., Bauce B. (2023). Desmoplakin Cardiomyopathy: Comprehensive Review of an Increasingly Recognized Entity. J. Clin. Med..

[76] Łajczak P.M., Jóźwik K. (2024). Artificial intelligence and myocarditis-a systematic review of current applications. Heart Fail. Rev..

[77] Haaf P., Buser P.T. (2017). Map to the future of cardiac magnetic resonance in myocarditis. Eur. Heart J. Cardiovasc. Imaging.

